# Monitoring Health Status: Development and Preliminary Validation of a Personal Health Index Using the International Classification of Functioning, Disability and Health

**DOI:** 10.2196/84802

**Published:** 2026-07-02

**Authors:** Ilkka Rautiainen, Lauri Parviainen, Veera Jakoaho, Sami Äyrämö, Jukka-Pekka Kauppi

**Affiliations:** 1 Faculty of Information Technology, University of Jyväskylä, PO Box 35, Jyväskylä, Central Finland, 40014, Finland, 358 142601211; 2 David Health Solutions Ltd., Helsinki, Finland; 3 Wellbeing Services County of Central Finland, Jyväskylä, Finland

**Keywords:** personal health index, International Classification of Functioning, Disability and Health, ICF, health informatics, data integration, health data standardization, longitudinal health monitoring

## Abstract

**Background:**

Effective health monitoring is essential for personalized care and comprehensive health assessment. Personal health indices and profiles offer a concise summary of an individual’s overall health, supporting both clinical decision-making and self-management. However, global standardization remains challenging due to diverse practices and data formats across countries.

**Objective:**

This study aimed to present a novel model for computing a personal health index and health profile using the International Classification of Functioning, Disability and Health (ICF) framework. The model was designed to handle incomplete and heterogeneous datasets and aimed to provide standardized, interpretable health metrics.

**Methods:**

We developed a recursive algorithm that calculates the health index based on the hierarchical structure of the ICF, using all available measurements. The model incorporates time decay and linkage reliability to weight input data. Preliminary validation was conducted on data from 505 individuals, using statistical correlation analyses with self-assessed health measures (EuroQol Visual Analogue Scale and pain ratings), and a sensitivity analysis was performed to assess model robustness.

**Results:**

The computed health index showed moderate positive correlations with EuroQol Visual Analogue Scale scores (all *P*<.001) and negative correlations with maximum pain trajectories, supporting its validity. Sensitivity analysis confirmed predictable behavior in response to input changes, and the model demonstrated resilience to missing data.

**Conclusions:**

The proposed model offers a flexible and scientifically grounded approach to computing personal health indices and profiles within the ICF framework. It enables the integration of diverse health data sources and supports the visual representation for clinical and personal use. This model has potential applications in health monitoring, rehabilitation planning, and machine learning–based health informatics.

## Introduction

### Background

Effectively describing and monitoring a person’s health is crucial for tailoring appropriate treatments. Comprehensive information about a person’s health enables targeted interventions and provides metrics that emphasize overall health status rather than isolated symptoms.

A personal health index aims to condense a person’s overall health into a single numerical value [1]. While the index seeks to summarize health in a singular number, a health profile represents health status through a set of scores [1]. These indices or profiles serve diverse purposes, offering a quick overview of an individual’s health status, beneficial for both health care professionals and individuals themselves. This systematic and comprehensible monitoring approach facilitates a broad perspective on health [1].

Given the multifaceted nature of health, summarizing it into a single value or profile requires a framework that can accommodate diverse and interrelated factors. A singular value from a health index can offer a quick overview of a person’s overall health, which is particularly useful in both clinical and self-monitoring contexts [1]. Additionally, exploring various aspects of health offered by a health profile is essential for a more detailed understanding of an individual’s health.

Capturing an individual’s overall health concisely can be challenging due to the multifaceted factors influencing health and its changes. Prevention, evaluation, treatment planning, and rehabilitation, especially in cases such as low back pain, necessitate consideration of psychosocial and social aspects [2]. It is, therefore, necessary to systematically describe all relevant aspects affecting a person’s health in a structured and standardized manner, presenting this information in an easily interpretable format.

Global standardization of health status assessment procedures for health index construction is challenging due to differing standards and practices across countries. Variations in treatment procedures, driven by diverse practices among countries, clinics, and therapists, hinder straightforward concatenation of datasets from separate clinics, especially with the use of different health questionnaires and languages.

Existing health indices often rely on fixed questionnaires or population-level parameters, limiting their adaptability and comparability across contexts. To address these limitations, a universally accepted framework is needed—one that can integrate heterogeneous data and support individualized assessment. In this study, we propose using the International Classification of Functioning, Disability and Health (ICF) [3], developed by the World Health Organization (WHO), as the foundation for constructing a personal health index. The primary goal of the ICF is to establish a standardized language and framework for describing health and health-related states [4]. It serves as a complement to the *International Classification of Diseases* (ICD) diagnosis classification system [4].

The ICF integrates 2 conceptual paradigms of disability—the medical and social paradigms. In the medical paradigm, disability is viewed as a health condition requiring medical care, often stemming from diseases, trauma, or other health conditions. On the other hand, the social paradigm considers disability as a socially created problem necessitating a political response. The ICF, by merging these paradigms, adopts a *biopsychosocial* approach, synthesizing biological, individual, and social aspects of health [4,5].

By leveraging the ICF’s comprehensive structure, our model aims to translate this biopsychosocial perspective into a quantifiable health index that can be used in both clinical and research settings. This model has the potential to shift from the current disease-based care model toward a *healthy aging* approach. The WHO’s concept of intrinsic capacity reflects a similar emphasis on monitoring functional trajectories across key domains to enable proactive, personalized interventions [6,7]. This approach aims to support a proactive, functioning-based way of tracking individual trajectories [3]. However, despite the clear advantages of the ICF, its practical implementation in clinical practice has proven challenging in various studies [8-11]. We argue that the health index framework introduced in this study also holds promise for simplifying the use of the ICF.

Unlike data-driven models that rely on statistical learning from large datasets, such as machine learning (ML) models that require training and may be prone to overfitting, the proposed health index is computed using a knowledge-driven, deterministic algorithm. This approach is grounded in the hierarchical structure of the ICF and applies predefined rules to aggregate available ICF-coded measurements. It operates independently of any training data or parameter estimation, ensuring consistency, interpretability, and robustness across diverse datasets. As a result, the model can be applied to any ICF-compatible data without modification, making it suitable for scalable and standardized health assessment.

Using the ICF framework involves converting original measurements to new variables in *ICF space* using accepted linking procedures. Throughout the paper, these variables are referred to as ICF codes according to ICF terminology. This approach facilitates the standardization of potentially heterogeneous datasets from different individuals into the same data space.

The health index is then recursively computed from the ICF tree structure using available measurements. Importantly, this computation does not require measurements from every node of the ICF tree, ensuring robustness to missing values. Additionally, if measurements do not cover all relevant health aspects, these gaps can be presented with the health index and health profile, and they can optimally be addressed in future data collection. Once the overall health index value is computed, it becomes possible to explore a health profile—that is, values for each ICF code separately. This approach provides insights into specific sectors of functioning, disabilities, and contextual factors affecting health. By adopting this comprehensive approach, we obtain a nuanced picture of an individual’s health, highlighting specific areas that may need improvement. By leveraging the health index, health profile, and the ICF, we can construct a clinical tool that facilitates cross-country data comparison and enables the development of a universal health metric.

In the context of ML, the proposed health index provides a framework for integrating datasets from diverse sources, thereby streamlining data preparation. The importance of maintaining uniform variables across all individuals cannot be overstated, as it is crucial for the development of robust ML models. A lack of standardization can result in smaller subsets, which may compromise the effectiveness of predictive models. Furthermore, the health index can serve as an optimization target in ML, facilitating the use of standardized datasets for predicting individual health outcomes. This could, for example, assist in the selection of personalized rehabilitation pathways. To our knowledge, this study marks the first usage of the ICF framework in forming a personal health index. In the following sections, we present the theoretical foundation, algorithmic implementation, validation results, and implications for clinical practice.

### Prior Work

Numerous health indices have been proposed in the literature. This section provides an overview of existing health indices and profiles, with an emphasis on research conducted in the 2000s. A literature search using Google Scholar was conducted in 2023 to identify models that explicitly used the term “health index” within this time frame. The focus was on individual-level health assessment studies, ensuring that the review reflects approaches relevant to personal health rather than population-level metrics.

One of the most straightforward methods for computing a personal health index involves the direct summation of responses to health-related questions. Kubik et al [12] constructed their personal health index by asking 6 questions on a Likert-type scale, ranging from 1 (strongly disagree) to 4 (strongly agree). These questions encompassed an individual’s perception of overall health, dietary patterns, and level of physical activity. The resulting health index score ranged from a minimum of 6 to a maximum of 24. Similarly, Frank et al [13] used a comparable summation method by querying participants about their smoking, drinking, exercise routines, and dietary habits to formulate a personal health index. Another simple approach, as demonstrated by Gallup [14], involved a personal health index derived from 5 yes-or-no questions concerning self-perceptions of health. These questions addressed general health problems, feelings of well-restedness, presence of physical discomfort, experiences of worry, and feelings of sadness. The resulting index was computed as the mean of all valid responses multiplied by 100, making the index range from 0 to 100.

Meijer et al [15] introduced a health index with an aim to be internationally comparable. Experts identified 25 variables measuring health and functional ability for inclusion in the index. The included variables consisted of reports of limitations with 10 mobility-related activities, arm and fine motor functions (such as walking, sitting, and reaching), 6 severe limitations with activities of daily living (such as dressing, bathing, and eating), and 7 less disabling problems in instrumental activities of daily living (such as preparing hot meals, making phone calls, and taking medication). Additionally, self-reported health and grip strength were incorporated into the index. The computation of this health index involved fitting the data with a special case of the LISCOMP model, integrating factor analysis and regression models. The health indices were reported to be comparable across different countries but were gender-specific. The primary focus of the study was centered more on examining the population-level health indices across various countries rather than discussing individual-level health indices.

Yi et al [16] developed 4 distinct health indices—namely, cardiovascular, stress, obesity, and management—derived from responses to specific questionnaires and objective measurements. These indices were constructed using weights assigned to each questionnaire item by experts in the field. The cardiovascular and stress indices were computed from a balanced combination of questionnaire responses and objective measurements, whereas the obesity index relied solely on measured data. The management index was formulated from questionnaire responses focusing on lifestyle and dietary habits. To consolidate these individual indices into an integrated health measure, the researchers weighted each element by the inverse of its corresponding standard deviation and summed the values together to create a final integrated health index.

Kohn [17] used multiple correspondence analysis (MCA) to construct a health index. MCA has similarities with principal component analysis (PCA) but is more suitable for discrete variables. The first dimension of MCA was reported to capture 80.4% of the principal inertia, corresponding to the variance in PCA. To facilitate interpretation, the analysis primarily focused on this first dimension, which represented the health index in this context. The range of observed continuous values for the health index was from 1 to 9, with higher values denoting better health. The selected domain of variables encompassed self-assessed health, a 36-point index evaluating mental and emotional health, reported health problems, disability status, a categorical number of accidents, and current smoking status. The suggested index allowed some flexibility for the index user, offering choices such as determining which domains to include when applying MCA for the index creation process.

Poterba et al [18] used PCA in creating the health index. The PCA for this purpose has been used since at least the 1980s [19,20]. In their approach, they used the first principal component as their health index. To aid the presentation, the “raw” health scores were converted into percentile scores for each respondent at different ages. The PCA was computed based on responses to 27 health-related questions, including activities of daily living (eg, difficulties in walking, lifting, or sitting), medical history (eg, experiences with stroke, diabetes, or cancer), self-reported health, BMI, and other relevant information.

Kim et al [21] suggested a toilet-based system for collecting data, such as pulse, blood pressure, oxygen saturation, BMI, electrocardiogram readings, and other vital signals. These data were used to construct 5 separate health indices—namely, heart, blood, fitness, muscle, and mental health index. Each index was derived by summing the respective normalized values together. Total health index represented the combination of all the 5 health indices, although its computation was not described in detail.

Chen et al [22] developed MyPHI, a method for constructing a personal health index. Using soft-label optimization, they created a unique prediction model for each disease category, handling infrequent and sparse data, and prioritizing recent health records. The method outperformed linear support vector machine and logistic regression models in their comparative analysis. Data from geriatric medical examinations, including patient profiling, laboratory tests, physical and external examinations, and mental health questionnaires, were used. Disease categories, derived from a cause of death dataset based on the ICD diagnosis classification, included lung, heart, cerebrovascular diseases, diabetes, stomach, colon, liver, pancreas, septicemia, and hypertension. The method generated a vector of scores reflecting personal health risks across disease categories. According to McDowell’s [1] definition, the output could be described as a health profile or a disease index, given its focus on predicting disease-specific risks.

Lai et al [23] introduced a novel personal health index based on the *technique for order preference by similarity to ideal solution*, an established ranking method from 1981, coupled with an independent entropy weighting approach. Additionally, the study used tensor decomposition to address problems stemming from missing data in health examination records. The authors highlighted the necessity for a more holistic approach to health assessment, acknowledging the multitude of variables collected during health examinations and difficulty in evaluating individual health status through only a small subset of these variables. They, however, selected 9 key health indicators in collaboration with domain experts, forming the basis of their health index. These indicators included systolic and diastolic blood pressure; BMI; total, high-density lipoprotein, and low-density lipoprotein cholesterol levels; fasting blood glucose; triglyceride levels; and thyrotropin. Each indicator held almost equal weight in the final computation of the health index.

The main characteristics of previous health indices and our index are summarized in Table 1. In the table, *“Individual adaptability”* refers to whether the model adapts to individual-specific data rather than relying solely on population-level parameters or fixed weights. A “*Yes*” indicates that the model can compute a personalized health index based on the available data for each individual. In some models (eg, MyPHI), this requires retraining or reconfiguring the model for each disease category or data type.

**Table 1.** Summary comparison of previous health indices and our index.

Study	Method	Data type	Individual adaptability	Supports missing data	Independent of training data
Kubik et al [12]	Summation of lifestyle questions	Questionnaire	No	No	Yes
Frank et al [13]	Summation of lifestyle questions	Questionnaire	No	No	Yes
Gallup [14]	Mean of yes or no questions	Questionnaire	No	No	Yes
Meijer et al [15]	LISCOMP model (factor analysis+regression)	Questionnaire+measurements	No	Yes	No
Yi et al [16]	Expert-weighted composite indices	Questionnaire+measurements	No	No	Yes
Kohn [17]	Multiple correspondence analysis	Questionnaire	No	Limited	No
Poterba et al [18]	Principal component analysis	Questionnaire	No	No	No
Kim et al [21]	Summation of normalized sensor data	Sensor measurements	No	No	No
Chen et al [22]	Machine learning with soft-label optimization	Clinical records+Questionnaire	No	Yes	No
Lai et al [23]	TOPSIS^a^+entropy weighting+tensor decomposition	Health examination records	No	Yes	No
This study	Recursive computation from ICF^b^ structure	Any ICF-compatible data	Yes	Yes	Yes

a TOPSIS: technique for order preference by similarity to ideal solution*.*

b ICF: International Classification of Functioning, Disability and Health.

Despite the variety of existing health indices, several limitations persist: lack of standardization and limited adaptability to heterogeneous data. Existing indices require retraining and/or manual configuration to accommodate new data types or disease categories. In contrast, the ICF-based personal health index proposed in this study is designed to automatically adapt to the available data for each individual without modifying the model, making it both flexible and standardized, and well-suited for scalable clinical use.

### International Classification of Functioning, Disability and Health

**Figure 1.**
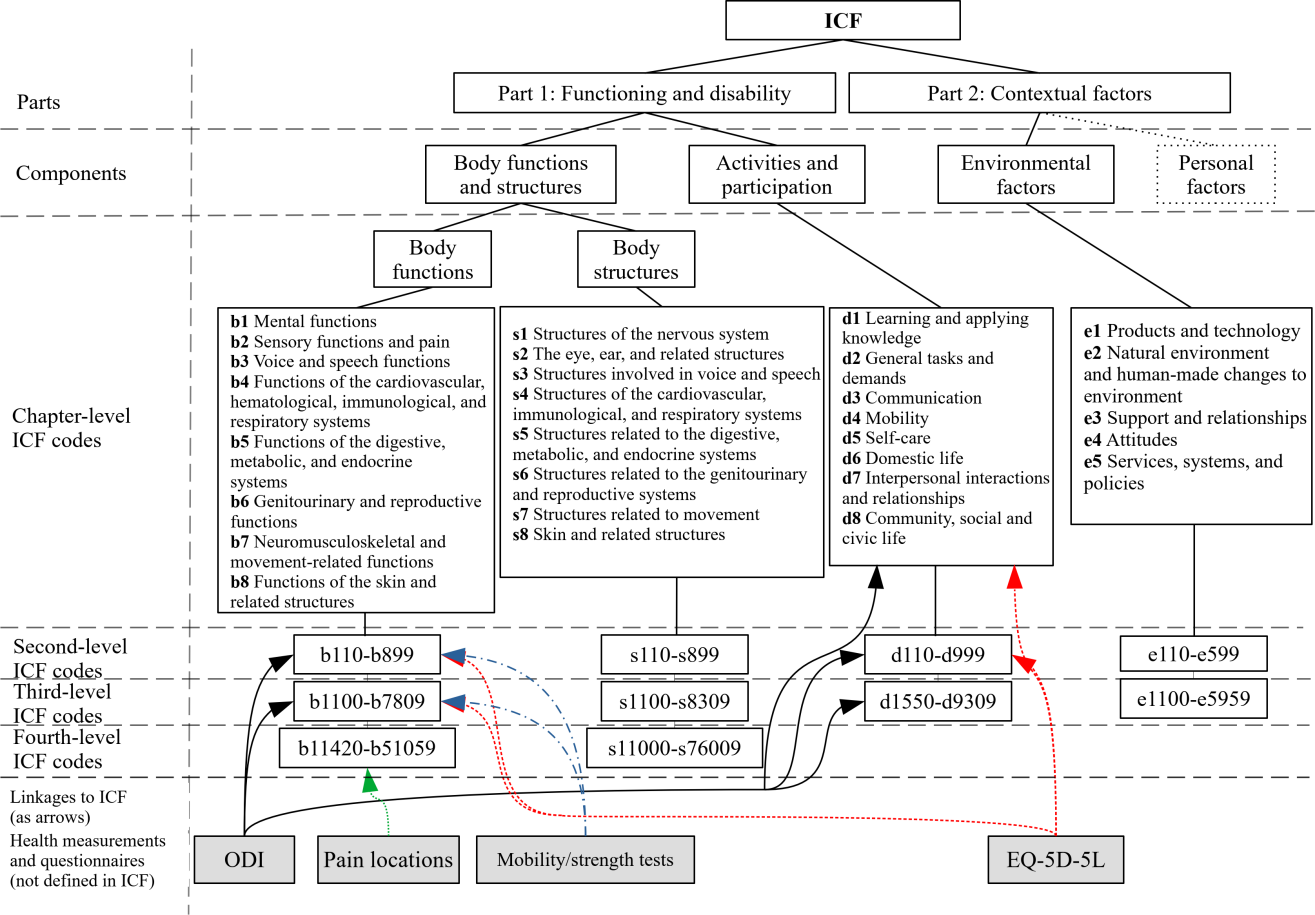
The hierarchical arrangement of the International Classification of Functioning, Disability and Health (ICF), compiled from data available in [3], along with the health measurements and questionnaires used during validation and their respective connections to the ICF.

Figure 1 provides an overview of the ICF structure. The 4 health measurements or questionnaires used and link to the ICF during the development and preliminary validation are depicted at the bottom of the figure, although they are not part of the ICF itself. Further discussion on these elements is presented in the section “Mapping data to ICF codes.”

The ICF classifies health and health-related problems into 2 main parts: *functioning and disability* and *contextual factors*. These are further divided into 4 main *components: body functions* (*b*) (physiological and psychological functions of body systems), *body structures* (*s*) (anatomical parts of the body), *activities and participation* (*d*) (execution of tasks and involvement in life situations), and *environmental factors* (*e*) (physical, social, and attitudinal environment). Although *personal factors* (background of an individual’s life) are part of the structure, they cannot currently be classified using the ICF [4].

The main components of the ICF encompass over 1400 subcategories distributed across 4 hierarchical levels. These categories exhibit a nested structure, where broader categories incorporate more detailed subcategories under the parent category. For instance, the ICF code *The eye, ear and related structures* (*s2*) in the *body structures* component (chapter 2) encompasses separate categories detailing structures such as the eye socket, eyeball, and surrounding areas [4].

The *chapter-level* ICF code is denoted by appending a single-digit number after the component *b*, *s*, *d*, or *e*, such as *b2* referring to the ICF code *sensory functions and pain*. The *second-level* ICF code uses a 3-digit number (eg, *b280* for *sensation of pain*). Moving further, the *third-level* ICF code consists of 4 numbers (eg, *b2801* for *pain in body part*), and the *fourth-level* code expands to 5 numbers (eg, *b28013* for *pain in back*) [4,5].

For classification completeness, an ICF code is accompanied by one or more *qualifiers*. These single-digit numbers signify attributes such as the magnitude of the level of health or the severity of the problem. A dot (.) serves as a separator between the ICF code and the qualifier(s). For instance, a complete ICF code with a qualifier could be *b280.1*, indicating a mild or slight *sensation of pain* [4].

All possible ICF codes support the first qualifier, although its application varies slightly among different main constructs. In *body functions* and *body structures* components, the first qualifier serves as a *generic qualifier*, signifying the extent or magnitude of an impairment. The generic qualifier can have values 0 (*no problem*), 1 (*mild problem*), 2 (*moderate problem*), 3 (*severe problem*), or 4 (*complete problem*). In the *activities and participation* component, the first generic qualifier indicates *performance*, representing a problem in the person’s current environment [4,5].

For *environmental factors*, the generic qualifier can be either a *barrier* or a *facilitator*, allowing positive contributors to be recorded. The negative scale is used similarly to the constructs mentioned earlier for barriers, whereas facilitators are indicated using a plus sign (+) as an ICF code or qualifier separator instead of a dot. For instance, ICF code *e145+2* indicates that products for education are a moderate facilitator, distinct from *e145.2*, which signifies that the products of education are a moderate barrier [4,5]. It is worth noting that facilitators are not part of our current health index computation model.

In addition to the first qualifier, additional qualifiers can be defined for *body structures* and *activities and participation*. For example, the nature of impairment can be described using the second qualifier in *body structures* (eg, *s7300.32* indicates a partial absence of the upper extremity) [4,5]. However, as our current computation model uses only the first qualifier, these additional qualifiers are not discussed further in this paper. Throughout the remainder of this paper, the term *qualifier* will exclusively refer to the first qualifier. In our proposed model, we introduce a minor addition to the ICF, which requires 2 attributes for the qualifier, specifically *linkage reliability* and *time elapsed* from the latest valid measurement. These attributes are discussed in the following sections.

## Methods

### Overview

In this section, we present the materials and methods used for computing the health index. Initially, we introduce the structure of the model used in the computation, outlining its fundamental components. Subsequently, we describe the overarching process involved in deriving the health index from the model. Following this, we delve into a more comprehensive explanation of the model elements, providing further insights into the ICF code–level computations.

### Materials

#### Data Collection and Linkage to ICF

For the initial evaluation of the health index, we used data collected from a singular David Health Solutions clinic, located in Helsinki, Finland, spanning from 2013 to 2019. David Health Solutions is a private health care organization with a global network of clinics, specializing in rehabilitation services. The dataset consisted of 505 individuals (mean age 48, SD 18 y; minimum 12 y, maximum 87 y; women: n=259; and men: n=246) undergoing rehabilitation treatment for diverse problems such as back, neck, hip, knee, shoulder, general health, and other unspecified reasons. Our original dataset included the following questionnaire and measurement datasets:

- Oswestry Disability Index [24]
- A generic health questionnaire EQ-5D-5L (EuroQol 5-dimensional, 5-level) [25]
- Mobility and maximal isometric strength tests using various spine concept rehabilitation machines
- Numeric pain rating scale for pain responses in different body parts [26]

A comprehensive overview of these questionnaires and measurements can be found in Multimedia Appendix 1. It is important to emphasize that we do not claim this to be the definitive optimal dataset; rather, it serves as an illustrative example of potential index usage. An essential preprocessing step in constructing the health index involves converting, or linking, variables from these original datasets to new variables known as ICF codes. The linkage of original variables to ICF codes is described in the following section.

#### Mapping Data to ICF Codes

The ICF linkage guidelines [27,28] advocate for the training of 2 medical professionals to establish linkages between the original data sources and the qualifiers of the ICF codes. During the linking process, 2 experts will independently create linkage information for each new data source. If their independent linkages match, they are accepted as final. In cases where discrepancies arise, a third expert opinion is sought to determine the definitive linkage.

Given the potential coexistence of scientifically validated and self-made (ie, expert derived but not formally validated) ICF linkages in the data, it is crucial to define separate linkage reliability values for different linkage types. Scientifically validated linkages, typically established through consensus procedures outlined in ICF-linking rules, can be assigned higher reliability scores, while nonvalidated linkages are downweighted to reflect their informal nature. Here, *r* ∈ [0,1] represents the *linkage reliability* designated for the corresponding source. The index user must specify the *r* value for every available source before computing the index. For instance, independently validated linkages may be assigned a maximum reliability of 1, whereas self-made linkages could be considered less reliable, resulting in *r* values typically set to less than 1.

For our dataset, formally validated ICF linkages do not exist, and therefore, we defined ICF linkages ourselves. While this led to lower linkage reliability when compared with formally validated linkages, this is not a limitation in this study because our focus is on the presentation of the new health index concept instead of proposing an optimal dataset for the health index computation. In fact, self-made linkages highlight the fact that the proposed health index concept is generic because it can be applied to heterogeneous datasets without requirements for the initial source and form of the data.

Details of the linkage for each questionnaire and measurement dataset are provided in Multimedia Appendix 1. For the EQ-5D-5L and Oswestry Disability Index questionnaires, data were available for 111 (22%) and 147 (29%) out of 505 individuals, respectively. Pain responses were accessible for 348 (69%) individuals, and mobility or strength test results were available for 420 (83%) individuals. Additionally, EQ-VAS, self-assessed health status on a scale from 0 to 100, was answered by 168 (33%) individuals.

The most frequently available ICF code in the dataset was *b780* (*sensations related to muscles and movement functions*), accessible for 420 (83%) individuals out of 505. The next 3 most common ICF codes available were *b7305* (*power of muscles of the trunk*), *b7355* (*tone of muscles of the trunk*), and *b7401* (*endurance of muscle groups*), all of which were available for 388 (77%) individuals. Codes related to pain localization (b28010, b28013, b28014, and b28015) were each available for 348 (69%) individuals, while b7302 was available for 321 (64%). Moderate availability was observed for b7300, b7350, and b7400, each present for 261 (52%) individuals. Codes b280, d450, and d5 were each available for 182 (36%) individuals. Several additional codes—b1340, d4103, d4104, d430, d470, and d910—were each available for 147 (29%) individuals, while b640 and d7702 were available for 136 (27%). The least frequently observed codes (b152, b1528, d230, d455, d510, and d540) were each available for 111 (22%) individuals. To be included in the above list, there had to be at least one measurement available for the specified ICF code.

#### Overview of Collected Data

There are 2 important aspects to explore in the data. First, the *duration* of a treatment period is defined as the number of full calendar days between the initial and final day of the treatment. When treatment occurs only once, the duration is considered 0. Second, we can assess the frequency or the number of treatment days during the treatment period, referred to as the *length* of the treatment sequence. For example, if an individual undergoes treatment for 3 weeks with 4 clinic visits, the duration of the treatment period would be 21 days, and the length of the treatment sequence would be 4.

**Figure 2.**
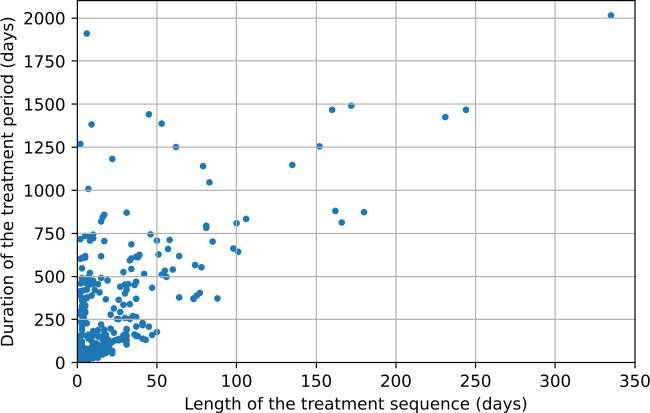
The duration of the treatment period and the length of the treatment sequence for each individual are presented. Most individuals are clustered in the lower-left corner, indicating relatively short treatments with few individual treatment days.

The median duration of the treatment period in the dataset was 69 days (IQR 0-301). Among individuals with nonzero duration, the median was 221 days (IQR 75-604). The median length of the treatment sequence was 3 days (IQR 1-16). Further exploration of this property is presented in Figure 2. The distribution of the length of the treatment sequence was as follows. A total of 148 (29%) out of 505 individuals had a sequence length of 1 day, indicating that for a substantial proportion of participants, data were available for only a single day. Furthermore, 61 (12%) individuals had 2 days, 44 (9%) had 3 days, and 26 (5%) had 4 days. Longer sequences were observed as follows: 71 (14%) individuals had 5 to 10 days, 48 (10%) had 11 to 20 days, 57 (11%) had 21 to 40 days, 31 (6%) had 41 to 80 days, 12 (2%) had 81 to 160 days, and 7 (1%) had 161 to 335 days.

#### Ethical Considerations

This study used previously collected and deidentified data from individuals who had provided informed consent for the use of their data in future research. The research did not involve any new interventions, physical or psychological risks, or other elements requiring ethical review as defined by the Finnish National Board on Research Integrity (TENK) guidelines on ethical review in human sciences [29]. Therefore, a separate ethical review was not required. The study was conducted in accordance with the principles of responsible conduct of research and data protection regulations. Personal data were processed in compliance with the EU General Data Protection Regulation, and deidentification was performed using procedures consistent with the Health Insurance Portability and Accountability Act Safe Harbor Method to minimize reidentification risk.

### Model Architecture and Components

**Figure 3.**
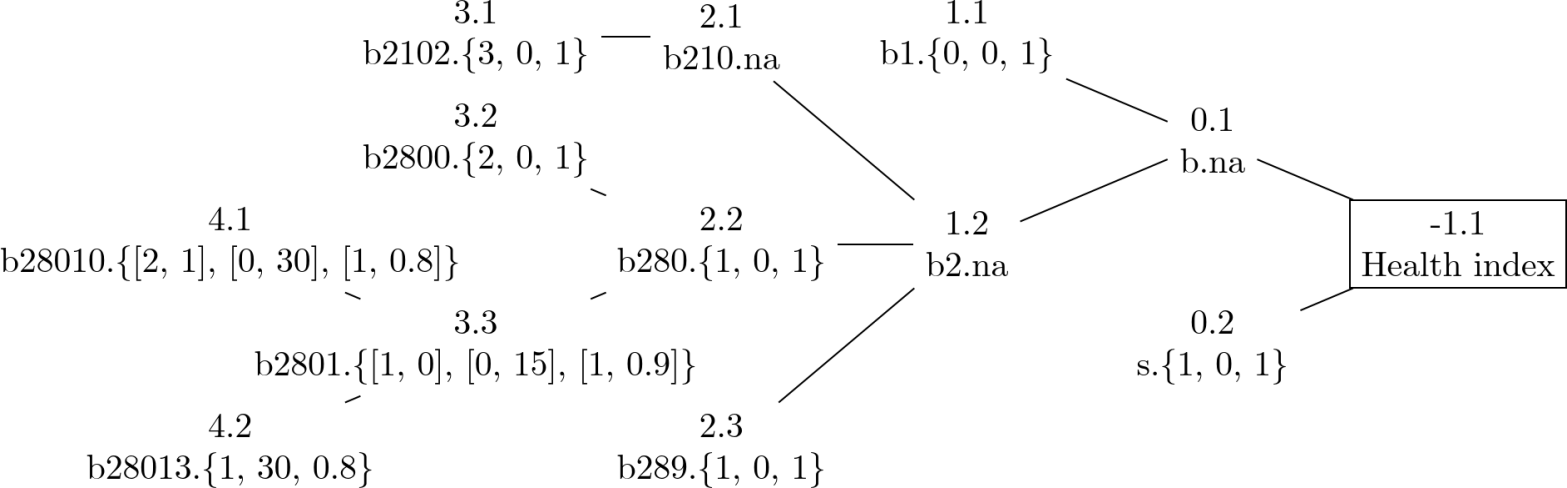
An artificial example of a tree structure containing International Classification of Functioning, Disability and Health (ICF) codes. Each ICF code is represented by 2 rows of information. The first row includes the level number and the ICF code number within that level, separated by a dot. The format used is level.index, where level corresponds to the ICF level (with level 4 being the deepest), and index represents the per-level running index. The ICF codes are listed in alphabetical order at each level, and the running index follows this order. The level labeled as 0 refers to the ICF top components (b, d, e, and s). Level 1 corresponds to the chapter-level ICF codes, level 2 to the second-level ICF codes, and so forth. The second ICF code row indicates the ICF code and 3 attributes associated with it—namely, ICF qualifier x, time elapsed from measurement (TE) (see Multimedia Appendix B in Multimedia Appendix 2 for details on time weighting), and reliability of the linkage r (see section “Mapping Data to ICF Codes” for details on item linkage). The format is ICF_code.{qualifier, TE, reliability}. When a qualifier is not available for the ICF code, na is indicated after the dot. For the sake of simplicity, measurements from the d and e components are not incorporated in the current example, despite being supported.

The proposed model structure aligns with the hierarchical organization of the ICF. Rather than incorporating all potential ICF codes, the model operates on whichever codes are available in a given dataset. In this study, we use the ICF codes listed in Figure 3 solely as an illustrative example of the model’s usage; the model itself is designed to be broadly applicable to any ICF-compatible data.

Within this restricted set of ICF codes, some codes may further be absent—meaning they were not measured for all individuals. Consequently, our model includes both observed codes (those with measurements) and empty codes (those without measurements). Notably, an ICF code within the model can be observed through multiple measurements. This scenario arises when multiple original data variables are linked to the same ICF code. Additionally, a single ICF code may have multiple measurements if linked to data recorded at different time points. Throughout this study, we refer to all linked measurements as *qualifiers*, consistent with ICF terminology.

For illustrative purposes, Figure 3 provides an example of a simplified tree structure containing ICF codes. As previously mentioned, multiple original measurements can be linked to the same ICF code, resulting in the observation of that ICF code through multiple qualifiers.

In Figure 3, we observe 2 ICF codes, each associated with 2 qualifiers, both linked to the same ICF code at the third and fourth levels. The first qualifier in the ICF code *b28010* (4.1) has a value of 2 (the first number inside the square brackets). Additionally, it has attributes—namely, time_elapsed=0 and reliability=1. The second qualifier, originating from a different data source, also corresponds to the same ICF code. It has a value of 1 (the second number inside the square brackets) and attributes—again, time_elapsed=30 and reliability=0.8. Furthermore, empty ICF codes are also included in Figure 3 for illustrative purposes.

It is important to recognize that the example in Figure 3 is simplified and does not incorporate data from the ICF components related to *activities and participation* (*d*) or *environmental factors* (*e*), although these components are indeed supported within the index. Furthermore, it is not common practice to assign measurements directly at the component level. However, in this example, we have made an exception with a qualifier assigned to the *body structures* component.

### Health Index Computation

The core concept behind our proposed approach is to systematically propagate information from the deepest nodes to the root of the tree. This process culminates in a single numerical metric known as the health index. Alternatively, if a health profile with multiple scores is desired, these metrics can be directly extracted from any level preceding the root level. We initiate the computation procedure at the leaf nodes of the tree. For instance, consider Figure 3, where we begin with ICF code *b28010* (4.1), then proceed to *b28013* (4.2), and subsequently to the next level represented by ICF code *b2102* (3.1), and so forth. This iterative computation continues until all ICF codes have been accounted for.

**Figure 4.**
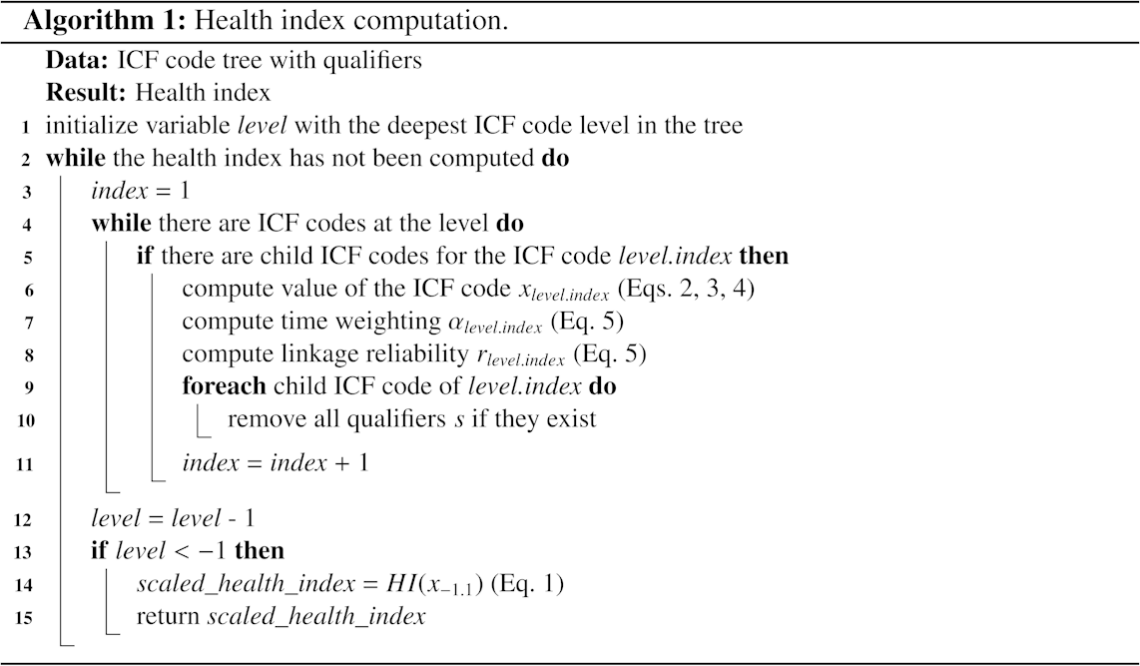
Health index computation algorithm. ICF: International Classification of Functioning, Disability and Health.

To compute the health index, we use a recursive algorithm that traverses the hierarchical ICF structure from the deepest nodes upward. At each level, the algorithm aggregates measurement values (qualifiers) using time decay and linkage reliability weights. This process continues until a single health index value is produced at the root level. The final computed value represents the unscaled health index, which we subsequently scale to a range of 0 to 100 to obtain the final health index. Detailed steps for this computation are outlined in the pseudocode presented in Figure 4, with the relevant equations provided following the algorithm. To streamline the algorithm presentation, some details that solely focus on performance optimization have been omitted. For example, given that the fourth level represents the deepest layer in the ICF framework, there is no practical need to verify child ICF codes at this level.

In essence, the algorithm ensures that all available data—whether recent or older or direct or indirect—is systematically weighted and aggregated. By processing the tree level by level, the model maintains consistency and robustness, even when data are missing or unevenly distributed. In the algorithm, the input data consist of an ICF code tree with qualifiers, similar to the one illustrated in Figure 3. The desired output is the health index. The procedure begins by initializing the variable *level* (line number 1) based on the deepest available ICF code level within the data. For instance, in the data represented in Figure 3, there are qualifiers associated with 2 ICF codes at level 4 (4.1 and 4.2). Consequently, the *level* variable would be initialized as 4 in this case. The lowest possible value for *level* is −1, corresponding to the root of the tree. At this root level, the final health index is computed.

The purpose of the outer *while* loop (lines 2‐15) is to sequentially process the ICF codes within the tree until the root is reached, at which point the health index is computed and returned. Within this loop, the variable *index* is initially set (line 3). The *index* serves as a per-level running counter, resetting to 1 whenever the tree level changes. For instance, in the processing order of ICF codes for the tree depicted in Figure 3, the sequence is 4.1, 4.2, 3.1, 3.2, 3.3, and so forth.

The inner *while* loop (lines 4‐11) serves the purpose of iteratively performing necessary computations until all the ICF codes at the current *level* have been processed. The first *if* statement (line 5) checks whether child ICF codes are available for the currently examined ICF code, denoted as *level.index*. For instance, in Figure 3, the child ICF codes of *b280* are *b2800* and *b2801*. When child ICF codes are available for a given ICF code, the computation process begins. In lines 6 to 8, we compute the value of the ICF code, denoted as *x_level.index_*, along with the time weighting *α_level.index_* and the linkage reliability *r_level.index_*. These computations are elaborated in detail in “Computing ICF code values and reliability.”

Subsequently, in lines 9 and 10, we remove all qualifiers from the child ICF codes associated with *level.index*. The purpose of this step is to ensure that these values are not reused in subsequent computations. Instead, we rely on the values computed during lines 6 to 8 as we continue the computation process. Additionally, the per-level running index (*index*) is incremented after handling each ICF code (line 11), whereas the level index (*level*) is decremented once all ICF codes at that level have been processed (line 12).

When the level index (*level*) drops below −1, it signifies that we have reached the root of the tree (line 13). At this point, we have computed the raw health index, denoted as *x*_-1.1_, according to the ICF code numbering scheme depicted in Figure 3. However, this raw value requires transformation to enhance its interpretability. Our desired presentation for the final health index is an integer ranging from 0 (indicating the worst health) to 100 (representing the best health). To achieve this, we invert the source and target values: whereas a raw health index value of 0 corresponds to optimal health, the same value within the target range signifies the poorest health condition.

As higher qualifier values indicate worse health, we invert the raw score to ensure that a higher health index corresponds to better health. This makes the final index more intuitive for clinical interpretation. The transformation is applied as follows, resulting in the final health index denoted as HI (line 14):


(1) $$HI(x_{-1.1})=nint\left(100-100\times \frac{x_{-1.1}-\min(x_{-1.1})}{\max(x_{-1.1})-\min(x_{-1.1})}\right),\#$$

where nint represents the nearest integer function, $x_{\text{-}1.1}$ is the raw value to be transformed, and $\text{min}\left(x_{\text{-}1.1}\right)$ and $\text{max}\left(x_{\text{-}1.1}\right)$ correspond to the minimum and maximum theoretical or actual raw values. Given that we know the theoretical minimum (0) and maximum (4) values for the raw health index, our primary approach is to directly scale and invert the raw values to fit our target range. Specifically, we define $\text{min}\left(x_{\text{-}1.1}\right)\text{=}0$ and $\text{max}\left(x_{\text{-}1.1}\right)\text{=}4$. The resulting value after this transformation (line 15) represents the final health index.

Subsequently, once the health index has been computed, the tree is reset to its original state. This entails removing all previously computed values from the ICF codes and restoring all qualifiers to their initial positions. This procedure ensures that any future computations do not inadvertently rely on previously computed values.

### Detailed Examination of Components

**Figure 5.**
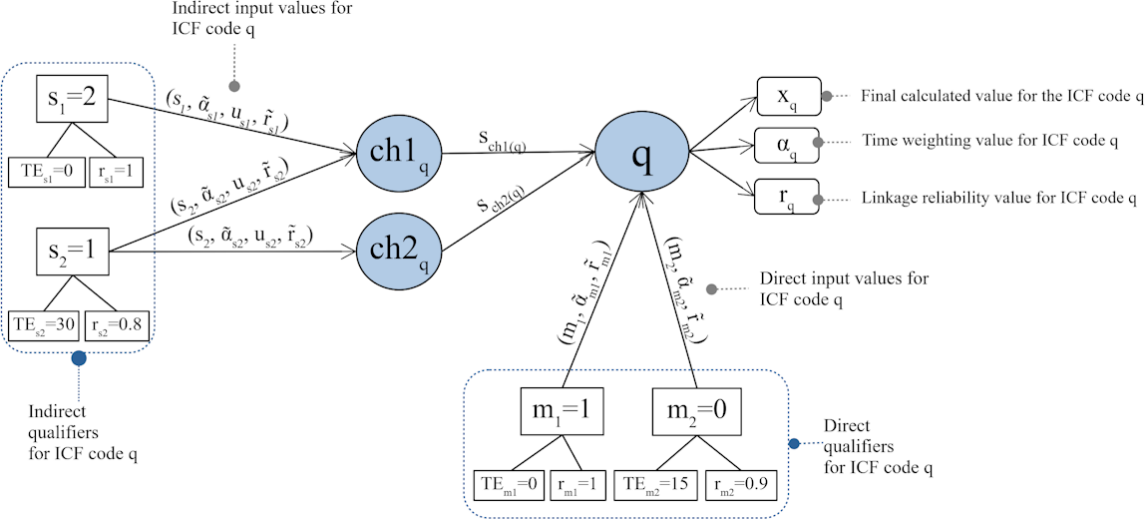
An International Classification of Functioning, Disability and Health (ICF) code–level example of the health index computation. In this figure, we have a viewpoint of an individual ICF code marked as q. Its child ICF codes are marked here as ch1_q_ and ch2_q_.

In Figure 5, we closely examine the components within our tree example. Specifically, we focus on 3 ICF codes: *b28010*, *b28013*, and *b2801*, extracted from Figure 3 for a comprehensive analysis. Each ICF code represents a specific health-related concept.

Let us denote the ICF code whose output value we compute as *q*. In Figure 5, this corresponds to node 3.3 (*b2801*) from Figure 3. Additionally, *q* has 2 child ICF codes: $ch1_{q}$ (equivalent to ICF code 4.1, *b28010*) and $ch2_{q}$ (equivalent to ICF code 4.2, *b28013*). These 3 codes are highlighted in blue color in Figure 5. Measurements mapped to these ICF codes are also shown in Figure 5 and are denoted as $m_{1},m_{2},s_{1},s_{2}\in \mathbb{Z}$. Owing to the ICF mapping performed as a preliminary step, values of these measurements are strictly between the range $0\leq m_{1},m_{2},s_{1},s_{2}\leq 4$, encompassing the range of qualifiers in the ICF. Separate notation is used for direct (*m*_i_) and indirect (*s*_j_) measurements from the point of view of the code of interest (*q*) to highlight the different roles of the measurements in the health index computation. The origin of the measurements is not important in the context of this example. We also illustrate that a single measurement (see S_2_ in Figure 5) can contribute to multiple ICF codes. The qualifiers play a crucial role in linking to specific ICF codes, as indicated by arrows. In this illustrative example, they are as follows:

1. A *moderate problem* (qualifier $s_{1}\text{=}2$) is recorded for ICF code *pain in head and neck* (*b28010*/$ch1_{q}$). The measurement is made on the latest available day in the treatment period, as indicated by $\text{T}{\text{E}}_{\text{s1}}\text{=}0$, and its linkage reliability is defined as the strongest possible ($r_{s1}\text{=}1$).
2. A *slight problem* (qualifier $s_{2}\text{=}1$) is recorded for ICF codes *pain in head and neck* (*b28010*/$ch2_{q}$) and *pain in back* (*b28013*/$ch2_{q}$). The measurement was made 30 days ago from the perspective of the latest available measurement, as indicated by $\text{T}{\text{E}}_{\text{s2}}\text{=}30$, and its linkage reliability is defined as ($r_{s2}\text{=}0.8$).
3. A *slight problem* (qualifier $m_{1}\text{=}1$) is recorded for ICF code *pain in body part* (*b2801*/*q*). The measurement is made on the latest available day in the treatment period, as indicated by $\text{T}{\text{E}}_{\text{m1}}\text{=}0$, and its linkage reliability is defined as the strongest possible ($r_{m1}\text{=}1$).
4. A *no problem* (qualifier $m_{2}\text{=}0$) is recorded for ICF code *pain in body part* (*b2801*/*q*). The measurement was made 15 days ago from the perspective of the latest available measurement, as indicated by $\text{T}{\text{E}}_{\text{m2}}\text{=}15$, and its linkage reliability is defined as ($r_{m2}\text{=}0.9$).
  The different components are marked in Figure 5:

- *Direct qualifiers (D) for ICF code q*: $m_{1}$ and $m_{2}$ are the *direct qualifiers* for ICF code *q*. In other words, they are directly linked to *q*. $\text{T}{\text{E}}_{\text{m1}}$ is the age of a qualifier measured in full days, and $r_{m1}$ denotes the reliability of the linkage.
- *Direct input values for ICF code q*: These values ($m_{i}$, ${\tilde{\alpha }}_{mi}$, ${\tilde{r}}_{mi}$) are associated with ICF code *q*. While the qualifier $m_{i}$ can be directly attached to the ICF code, other values must be computed based on the available data.
- *Indirect qualifiers (I) for ICF code q*: This component represents the 2 available qualifiers, $s_{1}$ and $s_{2}$, for the 2 child ICF codes $ch1_{q}$ and $ch2_{q}$. Qualifiers $s_{1}$ and $s_{2}$ serve as the *indirect* qualifiers for ICF code *q*. They are linked to *q* through its child ICF codes. The time elapsed value for $s_{1}$ is denoted by $\text{T}{\text{E}}_{\text{s1}}$, and its source linkage reliability is given by $r_{s1}$.
- *Indirect input values for ICF code q*: These values ($s_{j}$, ${\tilde{\alpha }}_{sj}$, $u_{sj}$, ${\tilde{r}}_{sj}$) are associated with the child ICF codes. They are used to compute the values $s_{ch1\left(q\right)}$ and $s_{ch2\left(q\right)}$ for the child ICF codes.
  There are 3 outputs in the diagram:
- *Final computed value for the ICF code q*: Denoted as $x_{q}$, this represents the ultimate value associated with ICF code *q*. It comprises both *direct* and *indirect* qualifiers. Specifically, $x_{q}$ is the weighted average of all available qualifiers: $s_{1}$, $s_{2}$, $m_{1}$, and $m_{2}$. In this case, it would be computed as follows: $x_{q}\text{=}\left({\tilde{\beta }}_{m1}^{D}m_{1}\text{+}{\tilde{\beta }}_{m2}^{D}m_{2}\right)\text{+}s_{ch1\left(q\right)}\text{+}s_{ch2\left(q\right)}\text{=}\left({\tilde{\beta }}_{m1}^{D}m_{1}\text{+}{\tilde{\beta }}_{m2}^{D}m_{2}\right)\text{+}\left({\tilde{\beta }}_{s1}^{I}s_{1}\text{+}{\tilde{\beta }}_{s2}^{I}s_{2}\right)\text{+}\left({\tilde{\beta }}_{s2}^{I}s_{2}\right)$, where β terms refer to weighting coefficients. Details regarding the equation are explained in the following section.
- *Time weighting value for q*: Denoted as $\alpha_{q}$, this value represents the weighted mean of all available time weighting α values, as defined in Equation 5.
- *Linkage reliability value for q*: Represented by $r_{q}$, this value is the weighted mean of all available *r* values, also defined in Equation 5.

### Computing ICF Code Values and Reliability

In the previous section, we explained the computation of ICF code values using a practical example for clarity. Now, in this section, we delve into a broader discussion on the computation of ICF code values and their associated reliability measures.

### Determining ICF Code Values

The value of the ICF code *q* is defined as follows:


(2) $$x_{q}=f\left(\sum_{i\in D}{\tilde{\beta }}_{mi}^{D}m_{i}+\sum_{k\in ch_{q}}s_{chk(q)}+\sum_{k\in ch_{q}}x_{chk(q)}\right)=f\left(\sum_{i\in D}{\tilde{\beta }}_{mi}^{D}m_{i}+\sum_{k\in ch_{q}}\sum_{j\in k}{\tilde{\beta }}_{sj}^{I}s_{j}+\sum_{k\in ch_{q}}{\tilde{\beta }}_{xk}^{I}x_{k}\right)$$

This definition consists of 4 separate elements:

1. Function f is a weighting function that can be selected to either give emphasis to higher qualifiers (exponential weighting) in data or to highlight lower qualifiers (logarithmic weighting). It is also possible to use no weighting at all (linear weighting). The different weighting functions are presented in more detail in Multimedia Appendix 2.
2. The first sum term $\sum_{i\in D}{\tilde{\beta }}_{mi}^{D}m_{i}$ represents the weighted sum of qualifiers $m_{i}$ directly linked to the ICF code *q*. Here, *D* denotes a set of *direct qualifiers*—that is, those qualifiers that are directly linked to *q*. Additionally, we introduce a total weighting term $\beta^{D}$ for direct qualifiers:

(3) $$\begin{array}{c} \beta_{mi}^{D}\text{=}{\tilde{\alpha }}_{mi}{\tilde{r}}_{mi} \end{array}$$

In this expression, α and *r* play essential roles. The first element, α, serves as a time weighting value that accounts for the age of the corresponding qualifier. Generally, as a qualifier ages, its influence diminishes in the computation. Further details on time weighting are discussed in Multimedia Appendix 2. The second element, *r*, corresponds to the linkage reliability defined in “Mapping data to ICF codes.” Notably, in Equation 2 and Equation 3, the terms β, α, and *r* carry the tilde notation ($\tilde{\beta },\tilde{\alpha },\tilde{r}$). This notation signifies that normalized values are used instead of raw values. A standard normalization procedure ensures comparability across different values. Refer to the Multimedia Appendix 2 on normalization for the 2 relevant equations.

1. The second sum term $\sum_{k\in ch_{q}}\sum_{j\in k}{\tilde{\beta }}_{sj}^{I}s_{j}$ represents the weighted sum of qualifiers $s_{j}$ that are indirectly linked to the ICF code *q* via its child ICF codes. Here, $ch_{q}$ refers to a child ICF code of *q* that possesses either a qualifier or a computed value. Consequently, each child ICF code of *q* contributes to the overall computation. The inner sum iterates through all the qualifiers associated with the child ICF code.
  Here, *I* denotes a set of *indirect qualifiers*. We introduce a total weighting term for indirect qualifiers, denoted as $\beta^{I}$. This term is a slightly modified version of the weighting term shown in Equation 3, defined as follows:

(4) $$\begin{array}{c} \beta_{sj}^{I}\text{=}{\tilde{\alpha }}_{sj}{\tilde{r}}_{sj}u_{sj} \end{array}$$

where $u_{sj}$ quantifies the *uniqueness of source* for the qualifier $s_{j}$. Specifically, it is computed based on the number of linkages between $s_{j}$ and child ICF codes of *q*. It is defined as $u_{sj}\text{=}1\text{/}z_{j},z_{j}\in N^{\text{+}}$, where $z_{j}$ represents the total linkages involving $s_{j}$ and child ICF codes of *q*. The purpose of introducing *u* is to adjust the qualifier weighting when the same source contributes to values for 2 or more ICF codes under the same parent ICF code. The terms α and *r* have the same purpose as in Equation 3.

1. The third sum term $\sum_{k\in ch_{q}}{\tilde{\beta }}_{xk}^{I}x_{k}$ mirrors the second sum term discussed earlier. However, there is a crucial distinction: it operates on the computed values (*x*) rather than the qualifiers (*s*). Notably, the second sum term becomes unnecessary here, as each child ICF code corresponds to a single computed value. Furthermore, the concept of uniqueness of source does not apply in this context. Consequently, we define ${\tilde{\beta }}_{xk}^{I}$ similarly to Equation 3, as ${\tilde{\beta }}_{xk}^{I}\text{=}{\tilde{\alpha }}_{xk}{\tilde{r}}_{xk}$.

### Assessing ICF Code Reliability

There are 2 values, $\alpha_{q}$ (equivalent to $\alpha_{level.index}$ in Figure 4 algorithm, line 7)—the weighted mean of time weighting α—and $r_{q}$ (equivalent to $r_{level.index}$ on line 8)—the weighted mean of linkage reliability *r*—that are computed for $x_{q}$. They are defined almost identically to $x_{q}$in Equation 2:


(5) $$\begin{array}{c} \alpha_{q}\text{=}\sum_{i\in D}{\tilde{\beta }}_{mi}^{D}\alpha_{mi}\text{+}\sum_{k\in ch_{q}}\sum_{j\in k}{\tilde{\beta }}_{sj}^{I}\alpha_{sj}\text{+}\sum_{k\in ch_{q}}{\tilde{\beta }}_{xk}^{I}\alpha_{xk} \end{array}$$

For computing $r_{q}$, we replace the α term in the equation with *r*. Here, the $\tilde{\beta }$ terms determine the weightings for each α or *r* component. These values, $\alpha_{q}$ and $r_{q}$, are attributes to the $x_{q}$ value and are intrinsically associated with it.

## Results

### Overview

In this section, we present the results of applying the proposed health index model to the David Health Solutions dataset. While some methodological details are included to support interpretation, the focus is on the outcomes of the validation analyses. The computed health indices for all individuals in the dataset underwent preliminary validation by comparing the results with self-reported EuroQol Visual Analogue Scale (EQ-VAS) responses and maximum pain ratings.

Two validation groups were established. The first group had a treatment period duration of at least 90 days, whereas the second group adhered to a 30-day limit. In the first group, the length of the treatment sequence (as described in “Overview of Collected Data”) was a minimum of 10 days. For the 30-day group, the treatment sequence length was at least 5 days. Individuals could be assigned to both groups, provided that the day and qualifier preconditions were met.

To explore the effect of different time weightings, we computed Pearson correlation coefficients for various time decay constants (γ). Multimedia Multimedia Appendix 2 provides a detailed definition of γ. The chosen values for γ were as follows:

· $\gamma_{1}\text{=}{\left(1\text{/}20\right)}^{1\text{/}30}\approx 0.905$ represented a very heavy time decay, where a 30-day-old qualifier contributed only 5% of its original time weight.
· $\gamma_{2}\text{=}{\left(1\text{/}3\right)}^{1\text{/}30}\approx 0.964$ represented our estimated preliminary potential time decay value in a real-life scenario. Practically, this value implies that a measurement made 30 days ago was given one-third of its original time weight.
· $\gamma_{3}\text{=}1$ signified no time decay at all.

The algorithm was implemented using the Python programming language [30]. Statistical analyses were conducted using SciPy [31], and figures were generated with Matplotlib [32].

### Comparing Self-Assessed Health Status Responses With Health Index

First, the health index was computed without emphasizing either lower or higher qualifiers. In Table 2, the EQ-VAS response was compared with the health index, and Pearson correlations are presented for both groups. We also explored the influence of changing the time decay constant (γ). The results predominantly reveal moderate positive correlations between the EQ-VAS response and the computed health index. The 90-day group consisted of 84 individuals, with a total of 125 EQ-VAS responses recorded. While most individuals had only one recorded EQ-VAS response, some provided 2, 3, or even 4 responses—all of which were used in the computations. The 30-day group included 115 individuals, with a total of 159 EQ-VAS responses.

**Table 2.** Pearson correlations for the EQ-VAS^a^ response versus health index^b^.

γ	30-d group	90-d group
${\left(\frac{1}{20}\right)}^{1\text{/}30}$	0.690	0.642
${\left(\frac{1}{3}\right)}^{1\text{/}30}$	0.700	0.659
1	0.654	0.599

a EQ-VAS: EuroQol Visual Analogue Scale.

b All reported correlations are statistically significant (*P*<.001).

### Comparing Maximum Pain Responses With Health Index

In the context of maximum pain versus the health index, we individually computed the Pearson correlation for each person between the maximum pain trajectory and the health index trajectory. We define maximum pain as the single highest value among the 4 self-assessed pain responses. Subsequently, a maximum pain trajectory was formed for each individual. As this information was also used in computing the health index, there was inevitably at least some correlation between the 2 values. However, this comparison provides a useful second perspective for model validation.

To prevent data from incorrectly showing significance, we applied the Bonferroni correction in the maximum pain versus health index trajectory correlation computations. Specifically, the α level was divided by *n*.

**Figure 6.**
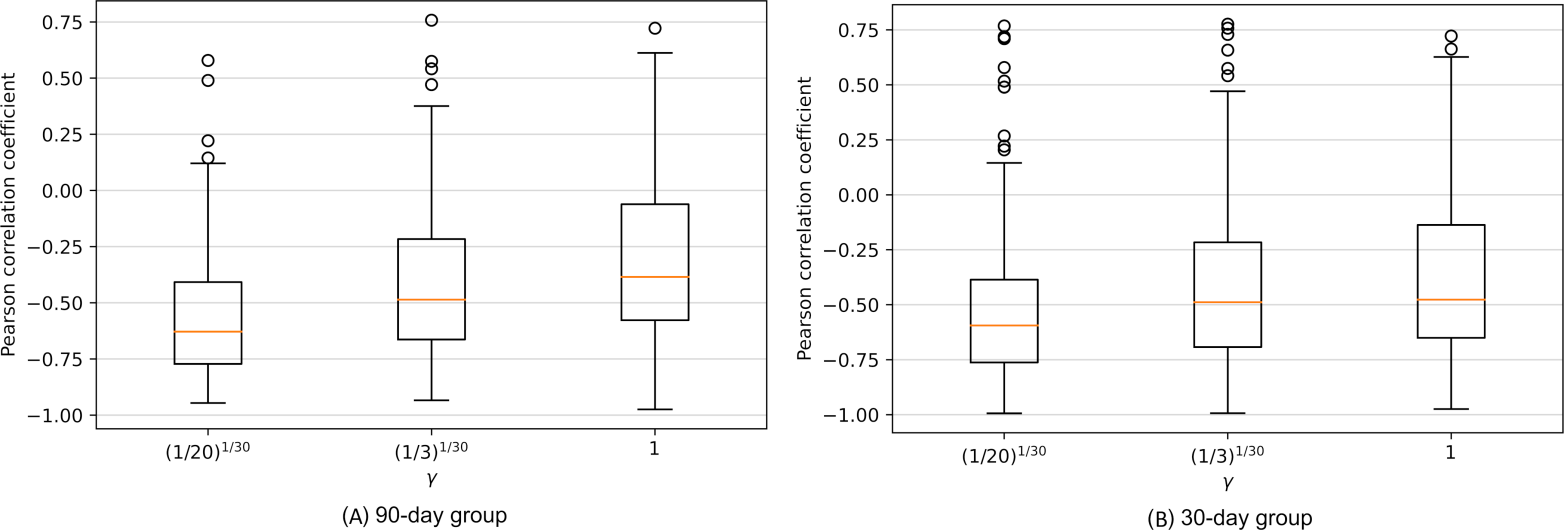
Boxplots illustrating Pearson correlations between the health index and maximum pain trajectories. (A) 90-d group, where the correlation medians for the 3 time decay groups (n=133) are −0.628, −0.486, and −0.385. (B) 30-d group, with correlation medians for the 3 time decay groups at −0.594, −0.489 (n=184), and −0.477 (n=182).

Figure 6 presents the distribution of correlation values for the 2 groups. Notably, a slight upward trend in median correlation is visible in both groups: as γ increases, the median correlation approaches zero. In all cases, the correlation was negative, varying approximately from low to moderate. There were small differences in the *n* values for the different groups. In some configurations, the health index value remained constant for certain individuals, making correlation computations impossible. These persons were omitted from the computations.

Examining the portions of significant correlations, we observed that for the 90-day group, Bonferroni-corrected significant (*P*<.05) portions were 55% for $\gamma_{1}$, 41% for $\gamma_{2}$, and 31% for $\gamma_{3}$. In the 30-day group, the similar portions were 41% for $\gamma_{1}$, 29% for $\gamma_{2}$, and 23% for $\gamma_{3}$. These findings suggest that as the length of the treatment sequence and duration of the treatment period increase, more significant correlations emerge.

**Figure 7.**
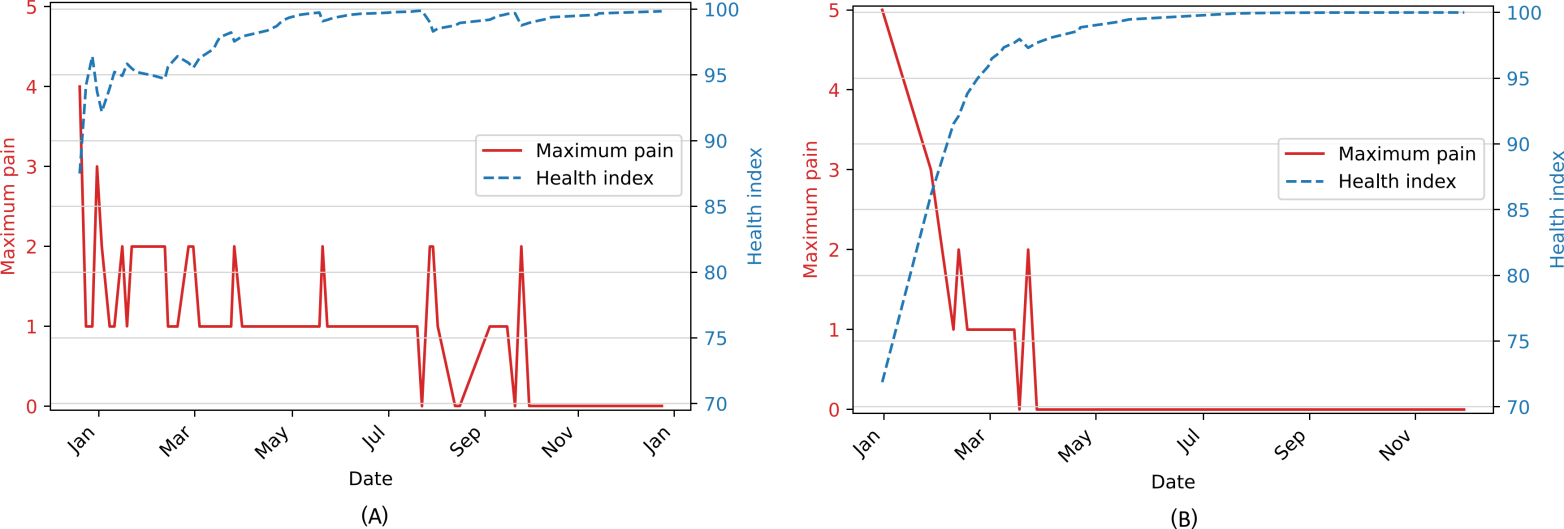
The maximum pain versus health index trajectories for 2 example individuals. The first case (A) demonstrates a moderate negative correlation with a treatment sequence length of 40 d. The second case (B) represents an extreme scenario with a very high negative correlation with a treatment sequence length of 28 d. Both cases share the same time decay value, γ_2_. Notably, the Bonferroni-corrected correlations were statistically significant (*P*<.001).

In Table 3, we further examine the effect of time series length by binning the maximum pain versus health index trajectories of individuals into 3 bins based on the length of the treatment sequence. The table presents both the 90-day group and the 30-day group, separated by a comma. These results also indicate that as more data points are included in the trajectory, the significant portion increases. Additionally, in Figure 7, 2 example individuals and their maximum pain versus health index trajectories are presented.

**Table 3.** Correlation statistics between maximum pain and health index, using a 3-bin approach based on treatment sequence length^a^.

Bin^b^	Day ranges	Bonferroni significant (*P*<.05) portions^c^	Median correlations	Subset *n*^d^
1	[10, 26], [5, 15]	18.8%, 7.9%	−0.385 (IQR −0.702 to −0.082),−0.569 (IQR −0.815 to −0.261)	48, 63
2	[27, 42], [16, 32]	37.5%, 18.3%	−0.508 (IQR −0.695 to −0.327),−0.376 (IQR −0.577 to −0.074)	40, 60
3	[43, 325], [33, 325]	68.9%, 65.6%	−0.509 (IQR −0.636 to −0.312),−0.511 (IQR −0.670 to −0.299)	45, 61

a The time decay value was defined as γ_2_.

b The 3 bins are approximately equal in size (tertiles). For each bin, we specify the range of treatment sequence lengths included. For instance, bin 1 encompasses individuals with treatment sequences lasting from 10 to 26 days (in the 90-d group) or from 5 to 15 d (in the 30-d group).

c Proportion of correlations that remain significant after Bonferroni correction at a 0.05 significance level.

d Number of individuals included.

### Impact of Nonlinear Weighting

In this section, we explore the impact of qualifier weighting on correlations. The model allows for the calibration of qualifier weighting by adjusting the parameter *y*. The weighting can operate under three distinct functions: (1) when y∈(0,2), the weighting is exponential, which implies that higher qualifier values carry more weight; (2) when y∈(2,4), the weighting is logarithmic, indicating that lower qualifier values are given more weight; and (3) when y=2, the weighting is linear, meaning that all qualifier values are treated equally, without any specific weighting. For details on value weighting, please refer to Multimedia Appendix 2.

**Figure 8.**
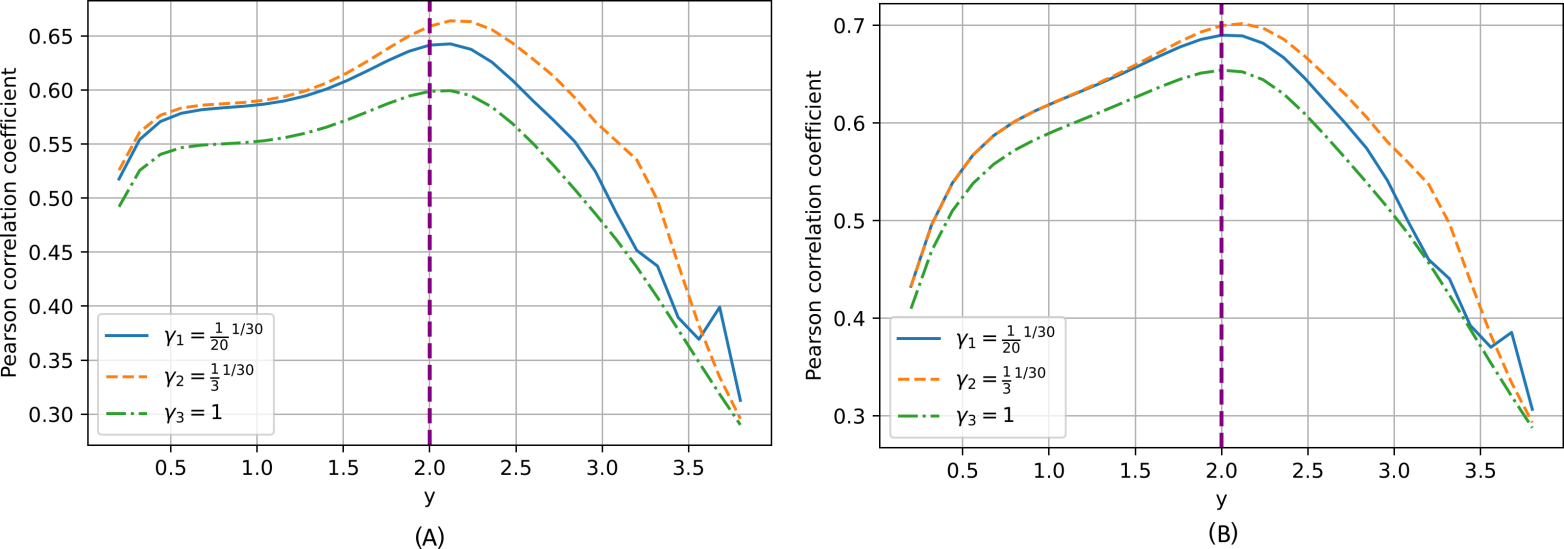
Pearson correlations between the health index and EQ-VAS response for 3 time decay constants. (A) 90-d group (n=125) and (B) the 30-d group (n=159).

In this study, we examined a total of 31 values for the *y* parameter. These values were selected to be evenly spaced on both sides of the linear case (y=2), ranging from y=0.2 to y=3.8 with intervals of 0.2. The results of the Pearson correlation between the health index and EQ-VAS response are depicted in Figure 8. For the 90-day group, the highest correlations for all 3 γ values were observed at y=2.12, with correlation values of 0.643, 0.664, and 0.599, respectively. In contrast, for the 30-day group, the highest correlations were observed at the linear weighting point (y=2). The correlations at this point are identical to those presented in Table 2.

**Figure 9.**
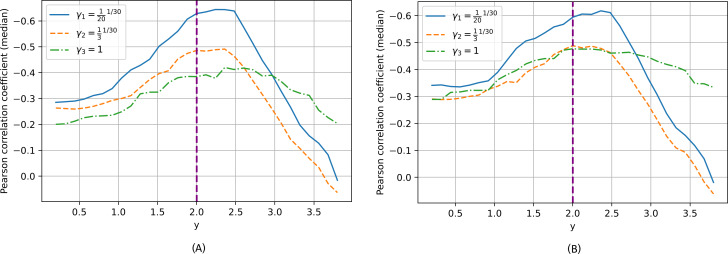
Median Pearson correlations between the health index and maximum pain for 3 time decay constants. (A) shows the 90-d group (γ_1_, γ_2_: n=133, γ_3_: n=132, 133) and (B) the 30-d group (γ_1_, γ_2_: n=184, γ_3_: n=181, 182, 183).

In the context of maximum pain versus the health index, the median correlations for both groups are depicted in Figure 9. For the 90-day group, the 3 γ values exhibited the highest negative correlations of −0.644 (at y=2.24), −0.491, and −0.420 (both at y=2.36), respectively. Conversely, the 30-day group demonstrated correlations of −0.618 (at y=2.36), −0.489, and −0.477 (both at y=2).

### Fundamental Sensitivity Analysis

The objective of conducting a sensitivity analysis is to understand how variations in input parameters influence the outcome of a model, in this case, the health index [33]. Our aim is to verify that the model behaves predictably and logically when modifications are made to the input variables. For instance, an increase in a single qualifier, while other qualifiers remain constant, should result in a decrease in the health index.

The ICF structure consists of over 1600 distinct ICF codes, each potentially having qualifiers. We use a simple data sample, as shown previously in Figure 3, to investigate the model’s response to a straightforward scenario: an increase or decrease in qualifier. In this scenario, we do not consider the reliability value (*r*) and assume it to be 1 for all qualifiers.

We target nodes that possess one or more qualifiers for value changes, with a particular emphasis on the qualifier and its impact on the final health index. For each targeted node, we explore all possible qualifiers ranging from 0 to 4, while maintaining constant values in other nodes, as illustrated in Figure 3. This setup produces 45 distinct health index outcomes, including the default.

The outcomes of the fundamental sensitivity analysis are detailed in Table 4. Throughout this analysis, the health index exhibited anticipated behavior. It is evident that ICF codes closer to the root (ie, the health index) of the tree exert a greater influence on the health index. Conversely, alterations in the most remote parts of the tree exert minimal influence on the overall health index. An additional characteristic worth noting is the time weighting. For example, alterations in the most recent qualifier, denoted as *b28010*_1_, exert a greater influence on the outcome compared to modifications in the qualifier from 30 days ago, represented as *b28010*_2_.

On the basis of the empirical patterns in this dataset, we propose 2 preliminary default parameters for future use: a moderate time decay value ($\gamma \text{=}{\left(\frac{1}{3}\right)}^{1\text{/}30}$) and a linear weighting function (y=2). These settings offered stable behavior and consistent correlations across both EQ-VAS and pain trajectories. They should be revisited when applying the model to other populations or more extensive ICF datasets.

**Table 4.** Outcomes of the health index from the sensitivity analysis. ICF codes b2801 (3.3) and b28010 (4.1) have 2 recorded qualifiers, each marked with a subscript of 1 or 2 to indicate their temporal sequence, with 3.3_1_ denoting the most recent qualifier. All other qualifiers were consistently fixed to the values shown in Figure 3. The time weighting, γ, was fixed at $(1/3{)}^{1/30}$

Qualifier^a^	S 0.2	b1 1.1	b280 2.2	b289 2.3	b2102 3.1	b2800 3.2	b2801_1_ 3.3_1_	b2801_2_ 3.3_2_	b28010_1_ 4.1_1_	b28010_2_ 4.1_2_	b28013 4.2
0	85.04	73.71^b^	74.59	75.34	82.74	75.46	74.09	73.71^b^	74.48	73.84	74.09
1	73.71^b^	67.94	73.71^b^	73.71^b^	79.73	74.59	73.71^b^	73.49	74.09	73.71^b^	73.71^b^
2	62.38	62.17^c^	72.83	72.08	76.72	73.71^b^	73.32	73.27	73.71^b^	73.58	73.32
3	51.05	56.40	71.95	70.44	73.71^b^	72.83	72.94	73.04	73.32	73.45	72.94
4	39.72	50.63	71.08	68.81	70.70	71.95	72.56	72.82	72.94	73.32	72.56

a Qualifier value (0‐4) used for each ICF code, ranging from 0.2 to 4.2 (refer to Figure 3 for ICF code identification),

b Default configurations.

c For instance, when the qualifier for ICF code b1 (1.1) is 2, the corresponding health index is 62.17.

## Discussion

### Principal Results and Comparison With Prior Work

In clinical practice, the health index could serve as a decision support tool for rehabilitation planning, chronic disease management, and progress tracking. For example, a physiotherapist could use the health index to track a patient’s recovery from back pain, identifying which functional domains are improving and which require further intervention. By providing a standardized and interpretable metric, it enables clinicians to quickly assess patient status, identify areas needing intervention, and monitor changes over time. The health profile further supports personalized care by highlighting specific domains of functioning and disability.

This study demonstrated that the proposed ICF-based health index correlates meaningfully with self-reported health measures. Moderate positive correlations with EQ-VAS and negative correlations with maximum pain trajectories support the index’s validity. Sensitivity analyses confirmed that the model responds predictably to changes in input qualifiers, with recent measurements exerting greater influence. The model also proved robust to missing data, successfully computing health indices across varying levels of data completeness. Furthermore, the impact of time decay and qualifier weighting parameters was systematically explored, revealing optimal configurations that enhance correlation strength.

From the existing research discussed in “Prior Work,” it is evident that the assessment of overall health status and the computation of health indices have followed varied approaches, using a diverse set of variables. Our approach to computing the health index and health profile is quite different from the methodologies discussed there. Below, we highlight significant divergences and commonalities between past methodologies and our approach.

**Figure 10.**
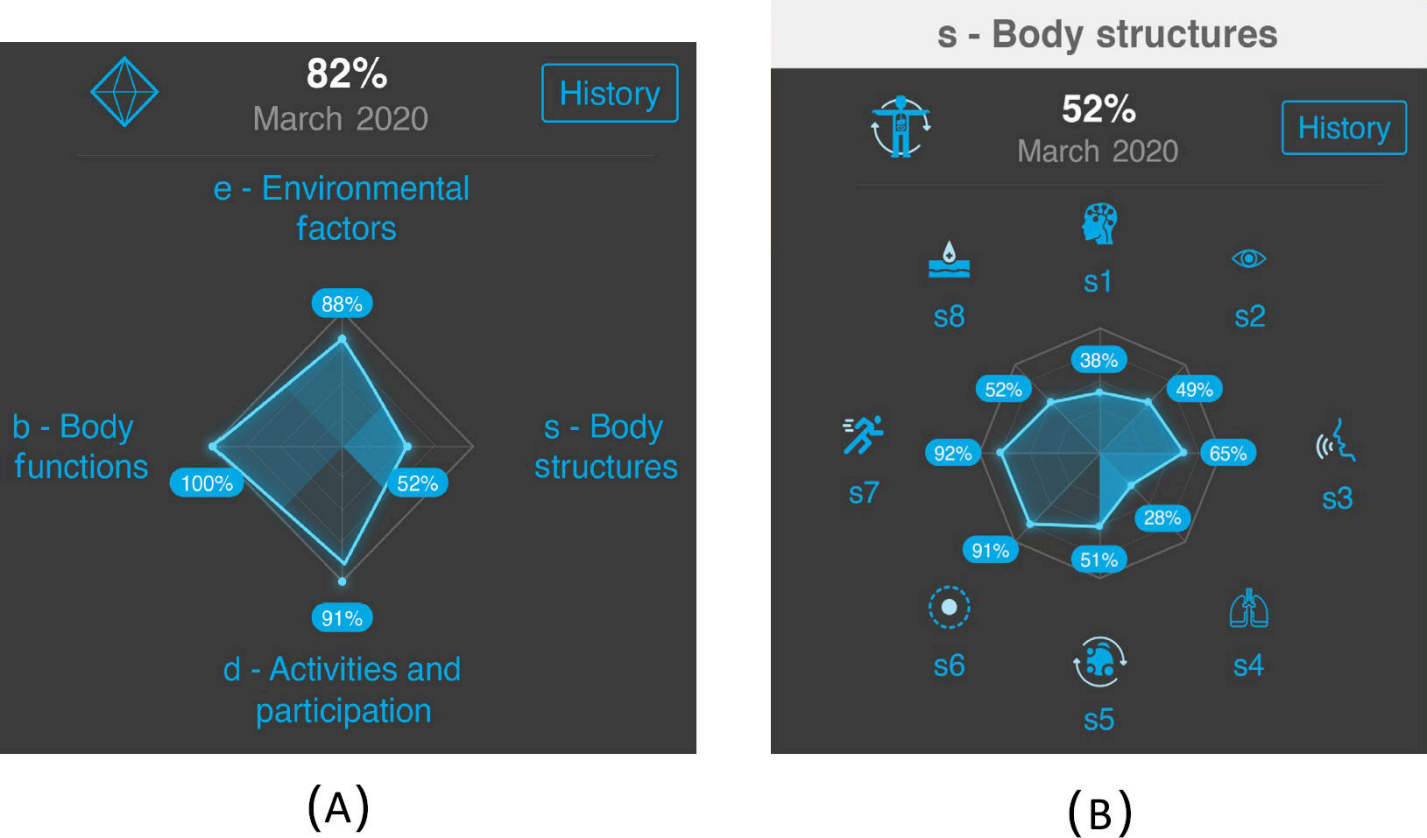
Preliminary graphical presentation of an individual's health index and health profile. A high-level view is shown in panel A, while panel B explores the health profile further in one of the components. In panel A, the overall health index, exemplified here as 82%, is displayed at the top. Additionally, the 4 main components of the International Classification of Functioning, Disability and Health are presented. These components constitute a basic-level health profile. Panel B provides a more detailed view into the body structures component, showcasing the chapter-level values and their respective coverages.

First, our proposed model exhibits a generic nature: it does not assume a predetermined set of variables for utilization. Instead, the model is based on the established ICF framework, enabling its adaptability to any dataset encoded in the ICF-compatible format. However, to ensure the derived health index comprehensively reflects overall health status, the set of ICF codes—forming the foundation of computation—should evidently encompass all aspects relevant to determining the health of the individual. This selection of ICF codes may vary individually; for instance, collecting specific information on low back pain might not contribute substantially if the individual does not experience this condition. Nevertheless, our proposed model maintains the capability to compute the health index across a spectrum, ranging from scenarios with very sparse data (where only one ICF code is available, for instance) to the theoretical extreme where all ICF codes are accessible. Deriving the health index from diverse datasets offers a unique advantage. However, this can potentially become a drawback if the characteristics of the data are not incorporated into the interpretation of the results. Visual inspection of the health index and health profile, along with information on how much data has been collected (called *coverage*), as exemplified in Figure 10, can aid in this process.

Furthermore, it is essential to highlight here that we do not prescribe any specific ICF sets for application; our preliminary validation using collected data represents just one potential usage. Notably, extensive research exists that defines ICF core sets outlining fundamental ICF codes crucial for various aspects related to functioning, disability, and health. For example, Cieza et al [34] defined a minimal generic set of domains for describing functioning and health for adults. Additionally, more specific core sets have been introduced, eg, for chronic conditions [35], including low back pain, diabetes mellitus, obesity, depression, and others.

Second, our proposed model operates independently from existing data—meaning that it does not rely on training data, coefficients, or predefined major model parameters. Instead, its core parameters stem directly from the hierarchical structure of the ICF, acting as weightings for different levels within this structure. Thus, one can compute the health index for an individual if there is some ICF-coded data available for that person. Moreover, the health index computed for an individual remains entirely distinct and unaffected by the health indices of others. In this aspect, our approach shares similarities with simpler summation-based methods (see, eg, [12-14]).

It is important to note the absence of a definitive *ground truth* for overall health status—there exists no singular, reliable response variable that could serve as a benchmark for validating any health index. In one study using ML [22], cause of death for specific diseases was used as the ground truth. Notably, our approach refrains from using ML elements, in part due to the absence of a definitive response variable for overall health. Furthermore, our aim does not involve predicting risks for specific diseases; instead, the model focuses on providing a concise estimation of health status. We argue that our model enables, through the use of ICF, a more holistic view on health, since by the WHO definition health is “not merely the absence of disease and infirmity,” but “is a state of complete physical, mental and social well-being” [36]. Moreover, aspects such as daily life activities, mental health, and social interactions best encapsulate most people’s intuitive notion of health [34,36], all of which the ICF is adept at capturing.

Finally, our proposed model is designed to handle the longitudinal aspect of infrequent health records and missing data fluently. These are important characteristics of health data [37]. However, only 2 studies (see [22,23]) considered these problems in their approaches. Nonetheless, we argue that our proposed methodology stands out for its simplicity and use of the inherent hierarchy within the ICF framework. Our model operates without expecting identical variables for different individuals. As the only prerequisite is to have the data in the ICF-compatible format, in fact, the whole concept of missing data is eliminated from the model’s perspective. Each individual’s data are processed independently, rendering the model robust against common health data problems. Consequently, the model generates output for any dataset meeting minimal requirements. Our objective is to give precedence to recent qualifiers in the computation of the health index, while still considering past qualifiers to a certain extent. This approach not only ensures a comprehensive view of the individual’s health status but also allows both experts and individuals to track the progression of various health-related trajectories over time.

Overall, existing research aligns with several aspects of our study objectives, notably in acknowledging disparities in data across various countries [15] and addressing challenges related to sparse, infrequent data with missing values [22]. However, existing health indices and profiles in previous studies primarily rely on predefined attributes for computing the final health index.

### Limitations

The ICF framework empowers its users to delineate the health and functioning of an individual in a highly detailed manner, beyond the first generic qualifier. In the *body structures* construct, the second qualifier can be used to indicate the nature of the change in the respective body structure, whereas the third qualifier specifies the location, for instance, left or right [4,38]. Moreover, in the *activities and participation* construct, the second generic qualifier (capacity) is used to denote limitations without assistance [4]. The proposed model, however, uses only the first generic qualifier, thereby not leveraging any additional information potentially provided by these other qualifiers.

In the context of the ICF’s *environmental factors* construct, the first qualifier can be used to signify either the positive effects (facilitators) or the negative effects (barriers) of the environment [4]. The current model is only equipped to handle barriers; hence, the potential positive impact of possible facilitators on the health index is not incorporated into the proposed model.

Qualifiers 8 (*not specified*) and 9 (*not applicable*) are not accommodated by the proposed model. At present, the values are treated as ordinal, and these nominal values cannot be used. This limitation might lead to a loss of some information, as these qualifiers are used in the ICF system to record information that cannot be captured using the [0,4] value range. Qualifier 8 should be applied when there is a problem, but the severity of that problem is unknown. Additionally, qualifier 9 is typically used in a situation when the use of the category is not appropriate for the individual [38]. These qualifiers have also been used in practical applications [39].

Taken together, these constraints mean that the current implementation captures only a portion of the information the ICF framework can offer. Expanding the model to incorporate additional qualifiers and environmental facilitators would allow it to reflect a broader range of clinically relevant information while keeping the computational framework unchanged.

A potential limitation concerning the practical usability of the model is that sparse data might provide a misleading impression about a person’s overall health. However, a basic data coverage metric can be used to assess the comprehensiveness of the data used and thereby also the reliability of the index outcome. Enhancing the coverage metric is also a key area for future improvements. The total coverage value could potentially be defined as a fraction representing the number of chapter-level codes that have associated measurements. To achieve a complete coverage of 100%, there would need to be a minimum of one measurement under each chapter-level code within the *body structures* component’s *s1* to *s8*, as well as all other chapter-level codes across the remaining 3 main components. In addition, as the ICF does not currently contain the classification of the personal factors, such as an individual’s age, they cannot be used in the current computation process. Moreover, the inclusion of personal factors has been identified as relevant and useful for the application of the ICF in different settings [40].

The dataset used for the preliminary validation of the health index is admittedly suboptimal, lacking the ability to encapsulate certain elements that define functioning. This study does not explicitly endorse the use of this particular set of ICF-coded data. Instead, we advocate for the use of datasets that offer a more comprehensive representation of all facets encompassed in the ICF for future applications. The existing ICF core sets can serve as a potential foundation for identifying the essential data required to collect for each specific condition. One possible solution would be to collect data that adheres to at least the minimal generic set proposed by Cieza et al [34], ensuring a baseline level of coverage across applications. An additional limitation in the preliminary validation dataset is that several variables were linked to ICF codes using expert-derived rather than formally validated linkages. Although these are downweighted using the linkage reliability parameter, formally validated linkages would further strengthen the results. The methodological framework itself is unaffected, but future applications may benefit from datasets where validated linkages are available. Incorporating formally validated or multirater ICF-linking procedures would improve reliability and external validity.

Another minor concern regarding the validation process is the current lack of differentiation between individuals under the age of 18 years and adults. The few individuals under 18 are incorporated into the larger group without any distinct consideration.

### Conclusions

We assert that the ICF framework is a solid foundation for developing a health index. As a globally recognized and standardized classification system, it provides a common language and conceptual basis for describing comprehensively factors influencing an individual’s health and functioning. Since its inception, the ICF has found extensive application in a variety of fields. These include, but are not limited to, clinical practice, policy formulation, social policy, and educational sectors [41]. Furthermore, it has become an indispensable tool in population health research projects worldwide [42].

An added advantage of the ICF is its compatibility with various questionnaires used to assess treatment response. These questionnaires have been linked to ICF codes, enabling the conversion of questionnaire responses into ICF code qualifiers. Many of these linkages have been scientifically validated, and comprehensive guides are available to establish new linkages for emerging datasets [28,38].

The proposed methodology consolidates information from various heterogeneous international sources, including interviews or anamnesis, validated questionnaires, physical tests, cognitive tests, etc, into a single, easily accessible location. This compatibility and consolidation process is crucial for advancing AI solutions. The training of ML algorithms requires identical variables from all individuals analyzed. Without dataset homogenization, only subsets of individuals and/or variables can be analyzed concurrently, reducing the predictive power of the trained models.

A single numerical value can be a useful tool for visualizing the individual’s condition before and after treatment and for tracking rehabilitation progress. However, to provide a more comprehensive view of an individual’s situation, we also aim to delve into the main categories at a more detailed level, rather than inferring a person’s functional capacity solely based on a single numerical value. Consequently, our proposed method is adaptable for computing a health profile. Within the ICF context, a basic health profile would likely include separate scores for each of the 4 ICF components: *body functions*, *body structures*, *activities and participation*, and *environmental factors*. These components can be further expanded for a more detailed examination of the elements within each category, providing comprehensive information about the individual.

Our goal is to make the health index and health profile highly illustrative, catering to both professionals and individuals undergoing treatment. If extensive data are recorded for an individual in the ICF, it can be challenging to assess and quickly understand the individual’s situation. The health index and health profile of an individual can also be presented visually, as shown in Figure 10, providing a concise overview of the individual’s condition.

Such visualizations can be integrated into electronic health records (EHRs) or patient portals, allowing both clinicians and patients to engage with the data. For clinicians, this supports efficient triage and treatment planning; for patients, it enhances understanding and motivation by making health status tangible and actionable. As the index is based on ICF codes, it can be integrated into existing EHR systems and used across institutions and countries, supporting interoperability and standardized health assessment.

The health profile allows for a deeper evaluation of specific domains of functioning, which can be particularly motivating by highlighting areas of improvement and those requiring attention. Previously, extracting and presenting such information was labor-intensive and often difficult for patients to interpret. The proposed model simplifies this process and improves accessibility. Additionally, a data coverage metric can help assess the completeness of the health profile, guiding clinicians in identifying gaps in assessment. In Figure 10, coverage is visually represented using transparency: low transparency indicates high data availability, while high transparency signals limited coverage. This visual cue supports informed decision-making and encourages targeted data collection to improve the reliability of the health index.

In current clinical practice, not all potential ICF subcategories are used. However, we continue to strive for a holistic understanding of an individual’s functional capacity. This is achieved through a combination of questionnaires, interviews, and tests, allowing us to gather extensive information, even if some ICF subcategories remain unfilled. For instance, when data from specific areas such as strength tests are entered into the system, it automatically assigns the appropriate ICF codes based on the results. Our proposed health index framework serves as a tool to standardize this process, thereby improving the efficiency and consistency of data collection and analysis in clinical settings.

### Future Work

Our ongoing efforts to enhance the index involve addressing the existing limitations outlined earlier. Specifically, we aim to improve the support for qualifiers beyond the initial qualifier and implement mechanisms to alert index users about insufficient data. Additionally, a step forward would be conducting a clinical validation of the index. Whereas the statistical analysis presented in this study provides an initial glimpse into the index’s functionality, a comprehensive clinical assessment will be needed to validate its practical utility.

A concrete example of the index usage is in the field of rehabilitation, where an iterative cycle based on the ICF, known as the Rehab-Cycle, has been developed for streamlined rehabilitation management [43]. This cycle describes different phases of rehabilitation: assessment, assignment, intervention, and evaluation. The use of the health index facilitates the generation of a compact numerical measure for both the preintervention assessment and postintervention evaluation in clinical practice. Furthermore, this index can assist in enhancing resource allocation and selecting appropriate interventions during the assignment and intervention phases. As a result, it allows for a more thorough evaluation of rehabilitation effectiveness and enables comparisons across various countries and rehabilitation methodologies.

Beyond rehabilitation, the health index has potential applications in broader clinical contexts. In primary care, it could support routine health assessments by providing a standardized snapshot of patient functioning. In geriatrics, it may help monitor age-related decline and guide interventions for healthy aging. Although intrinsic capacity (IC) was not directly assessed in this study, our approach aligns closely with the IC framework, and future work may explore how the proposed health index could support IC-oriented monitoring and proactive care models as outlined in recent WHO digital health initiatives. In mental health, the index could be used to track functional outcomes and support personalized treatment planning. Integration into EHRs or patient-facing platforms could enable real-time monitoring, enhance clinician-patient communication, and support shared decision-making.

With the prospective incorporation of ML techniques, the health index or health profile scores could serve as optimization targets within predictive models. This strategy allows for a comprehensive improvement of an individual’s health, rather than concentrating on a single health parameter. For instance, it could assist in determining the optimal rehabilitation pathway or treatment strategy for an individual. Additionally, the standardized nature of the index supports cross-institutional and international data harmonization, which is essential for scalable AI applications in health informatics.

## Supplementary material

10.2196/84802 Multimedia Appendix 1 Details on data linkages.

10.2196/84802 Multimedia Appendix 2 Details on defining weightings and normalization.

## Abbreviations

EHR: electronic health record
EQ-VAS: EuroQol Visual Analogue Scale
IC: intrinsic capacity
ICD: International Classification of Diseases
ICF: International Classification of Functioning, Disability and Health
MCA: multiple correspondence analysis
ML: machine learning
PCA: principal component analysis
WHO: World Health Organization
