# Supplementary material for: Monitoring Health Status: Development and Preliminary Validation of a Personal Health Index Using the International Classification of Functioning, Disability and Health
Source: JMIR Hum Factors. 2026 Jul 2;13:e84802. doi: 10.2196/84802 (PMC13337805; doi:10.2196/84802)
Supplement: Multimedia Appendix 1 [file humanfactors-v13-e84802-s001.docx]

Monitoring Health Status: Development and Preliminary Validation of a Personal Health Index Based on the International Classification of Functioning, Disability and Health

**Authors:**

Ilkka Rautiainen, Lauri Parviainen, Veera Jakoaho, Sami Äyrämö, Jukka-Pekka Kauppi

# Appendix 1. Details on data linkages

The Oswestry low back pain disability questionnaire (ODI) includes one item pertaining to pain and nine items related to activities of daily living (lifting, walking, social life, personal care, sitting, standing, sleeping, traveling, and sex life). Each item is scored on a scale of 0 to 5, with 5 denoting the highest level of disability ^1^. We established these and all subsequent linkages independently, using David’s in-house ICF experts. The ODI linkages are illustrated in eTable 1. Additionally, the translations between the original ODI responses and their corresponding values as ICF code qualifiers are presented in eTable 2.

eTable 1. The ODI questions, their purpose, and their equivalent ICF codes. The response options are not listed.

| **Item as appeared** | **Purpose of information  in question** | **ICF code(s)** |
| --- | --- | --- |
| Pain intensity | Level of pain | *b280* |
| Personal care  (washing, dressing etc.) | Pain related to personal care  tasks | *b280, d5* |
| Lifting | Pain related to lifting objects | *b280, d430* |
| Walking | Pain related to walking | *b280, d450* |
| Sitting | Pain related to sitting | *b280, d4103* |
| Standing | Pain related to standing | *b280, d4104* |
| Sleeping | Pain related to sleep | *b280, b1340* |
| Sex life (if applicable) | Pain related to sexual activities | *b280, d7702, b640* |
| Social life | Pain related to participation  in social activities | *b280, d910* |
| Travelling | Pain related to capacity for  travelling | *b280, d470* |

eTable 2. The original ODI responses and their translated equivalent values in the ICF system.

| Original ODI response: | 0 | 1 | 2 | 3 | 4 | 5 |
| --- | --- | --- | --- | --- | --- | --- |
| ICF code qualifier: | 0 | 1 | 2 | 3 | | 4 |

The five-level version of the EQ-5D generic health questionnaire, known as EQ-5D-5L, encompasses five questions addressing mobility, self-care, usual activities, pain/discomfort, and anxiety/depression. Each question is scored on a scale of 1 to 5, with 5 indicating the highest level of disability. In addition to these five questions, the questionnaire includes a self-assessed score for overall health, known as EQ-VAS, which is rated on a 0 to 100 scale. However, EQ-VAS is not utilized in the computation of the health index; it is solely used for the external validation of the index. Given that there are five response options for all questions, excluding EQ-VAS, similar to the 0 to 4 scale of the ICF qualifier, the responses can be directly mapped as ICF code qualifiers. The questions and their corresponding linkages to ICF codes are outlined in eTable 3.

Pain in the back, hip/leg, neck, and shoulder/arm regions were assessed using the numeric pain rating scale (NPRS) on a 0–10 scale at the onset of each clinic visit. Given the frequent measurement of these variables, it is crucial to incorporate these datasets into the analysis, in addition to the questionnaire data, which was typically collected only once or twice throughout the entire treatment period.

eTable 3. The EQ-5D-5L questions, their purpose, and their equivalent ICF codes. The response options are not listed.

| **Item as appeared** | **Purpose of information  in question** | **ICF code(s)** |
| --- | --- | --- |
| Mobility | Problems with walking | *d450, d455* |
| Self-care | Problems with self-care,  specifically washing and  dressing | *d5, d510, d540* |
| Usual activities (e.g. work,  study, housework, family  or leisure activities) | Problems with daily routine  / usual tasks | *d230* |
| Pain / Discomfort | Level of pain or discomfort | *b280* |
| Anxiety / Depression | Level of anxiety or  depression | *b152, b1528* |
| We would like to know how  good or bad your health is  TODAY. | General health perception | *N/A* |

eTable 4 presents the linkages between the original pain and the corresponding ICF codes. Additionally, eTable 5 illustrates the translations between the original pain responses and their equivalent values as ICF code qualifiers. For instance, an original pain response of ''3'' for back pain would translate to ICF code *b28013* as qualifier ''1''.

eTable 4. The original pain locations and their equivalent ICF codes.

| **Pain location** | **Corresponding ICF code** |
| --- | --- |
| Back | *b28013 (Pain in back)* |
| Hip/leg | *b28015 (Pain in lower limb)* |
| Neck | *b28010 (Pain in head and neck)* |
| Shoulder/arm | *b28014 (Pain in upper limb)* |

eTable 5. The original pain responses and their translated equivalent values in the ICF system.

| Original pain response: | 0 | 1 | 2 | 3 | 4 | 5 | 6 | 7 | 8 | 9 | 10 |
| --- | --- | --- | --- | --- | --- | --- | --- | --- | --- | --- | --- |
| ICF code qualifier: | 0 | | 1 | | 2 | | | 3 | | 4 | |

We also established new linkages for the mobility/strength tests. Strength and mobility levels concerning relevant spine functions were measured using David Health Solutions' spine concept rehabilitation machines and expressed in newton-meters and degrees, respectively. These values were then transformed into relative changes in comparison to the average values of a reference population. Superior mobility/strength than the reference was mapped to 0%, corresponding to *no problem*. The interventions implemented with the machines, as well as their ICF codes, are detailed in eTable 6. The translations of the original relative change values into the ICF code qualifiers are demonstrated in eTable 7. The values were subsequently converted to the ICF domain using the linkages depicted in these two tables. For instance, a relative change of 20% observed through the *120 Trunk Rotation* intervention would be linked with qualifier ''1'' to the ICF codes *b780*, *b7302*, *b7305*, *b7355*, and *b7401*.

eTable 6. The original spine concept rehabilitation machine interventions, their purpose and their equivalent ICF codes.

| **Intervention** | **Target of intervention** | **Corresponding ICF codes** |
| --- | --- | --- |
| 110 Trunk Extension | Increase of dorsal, lumbar and thoracic region muscle tone and strength, Pain prevention | *b7305 (Power of muscles of the trunk), b7355 (Tone of muscles of trunk), b7401 (Endurance of muscle groups), b780 (Sensations related to muscles and movement functions)* |
| 130 Trunk Flexion | Increase of abdominal region muscle tone and strength, Pain prevention |  |
| 120 Trunk Rotation | Increase of lateral and abdominal region muscle tone and strength, Pain prevention | *b7302 (Power of muscles of one side of the body),*  *b7305 (Power of muscles of the trunk), b7355 (Tone of muscles of trunk), b7401 (Endurance of muscle groups), b780 (Sensations related to muscles and movement functions)* |
| 150 Trunk Lateral Flexion | Increase of lateral and abdominal region muscle tone and strength, Pain prevention |  |
| 140 Cervical Extension / Lateral Flexion | Increase of cervical region muscle tone and strength, Pain prevention | *b7300 (Power of isolated muscles and muscle groups),*  *b7302 (Power of muscles of one side of the body),*  *b7350 (Tone of isolated muscles and muscle groups),*  *b7400 (Endurance of isolated muscles), b780 (Sensations related to muscles and movement functions)* |
| 160 Cervical Rotation | Increase of cervical region muscle tone and strength, Pain prevention |  |

eTable 7. The original spine concept rehabilitation machine relative changes (%) and their translated equivalent values in the ICF system.

| **Original relative change (%):** | $0 \leq x \leq4$ | $4 < x \leq24$ | $24 < x \leq49$ | $49 < x \leq95$ | $95 < x \leq100$ |
| --- | --- | --- | --- | --- | --- |
| **ICF code qualifier:** | 0 | 1 | 2 | 3 | 4 |

# References

1. Koç M, Bayar B, Bayar K. A comparison of Back pain functional scale with Roland Morris disability questionnaire, Oswestry disability index, and short form 36-health survey. *Spine*. 2018;43(12):877-882.
