# Supplementary material for: Monitoring Health Status: Development and Preliminary Validation of a Personal Health Index Using the International Classification of Functioning, Disability and Health
Source: JMIR Hum Factors. 2026 Jul 2;13:e84802. doi: 10.2196/84802 (PMC13337805; doi:10.2196/84802)
Supplement: Multimedia Appendix 2 [file humanfactors-v13-e84802-s002.docx]

Monitoring Health Status: Development and Preliminary Validation of a Personal Health Index Based on the International Classification of Functioning, Disability and Health

**Authors:**

Ilkka Rautiainen, Lauri Parviainen, Veera Jakoaho, Sami Äyrämö, Jukka-Pekka Kauppi

# Appendix 2. Defining weightings and normalization

The health index incorporates two distinct weighting elements: time decay weighting and various value weighting functions. The objective of time weighting is to prioritize more recent qualifiers. The utilization of value weighting functions offers the flexibility to adjust measured values, either amplifying the impact of higher disability levels or minimizing their influence on the data.

## Value weighting functions

The selection of the weighting function commences with the definition of a tuning parameter $y\in]0,4[$, which dictates the steepness of the weighting function. The curve always originates from the point $\left( 0,0 \right)$, is adjusted to intersect the point $\left( 2,y \right)$, and culminates at the point $\left( 4,4 \right)$. The weighting function space is visualized in eFigure 1. The chosen *y* value directly influences the type of function employed in the fitting process. There are three distinct function types.


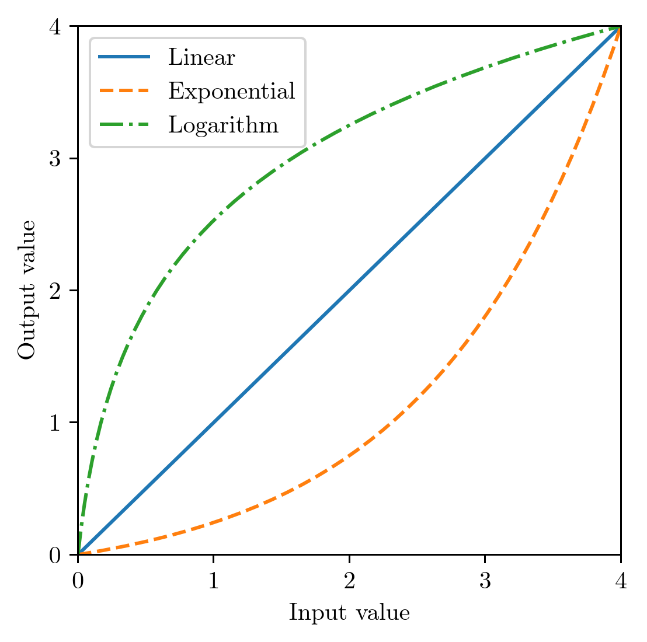


eFigure 1. Weighting functions visualized using example y values.

Firstly, the function is exponential when $y\in\left( 0,2 \right)$, defined as:

$$\begin{aligned} f\left( x \right)=ae^{bx}+c.\#\left( e1 \right) \end{aligned}$$

Secondly, the function is logarithmic when $y\in\left( 2,4 \right)$, defined as:

$$\begin{aligned} f\left( x \right)=a \text{ln}\left( bx+1 \right).\#\left( e2 \right) \end{aligned}$$

Finally, the function is linear when $y=2$. Linear function is simply defined as $f(x)=x$.

The values for *a*, *b*, and *c* in the exponential and logarithmic functions are determined during the standard curve fitting process. All weighting functions adhere to the range $x\in\left[ 0,4 \right]$, which aligns with the basic range of the generic qualifier in the ICF. The three weighting functions are illustrated in eFigure 1, utilizing values of $y=0.75$ for the exponential function and $y=3.25$ for the logarithmic function. Notably, the selection of the exponential weighting function implies an emphasis on higher values, i.e., higher disability levels, in the data. Conversely, the employment of the logarithmic weighting function results in an increase in all input values by the function, thereby emphasizing lower disability levels.

## Time weighting

Upon linking the data to the ICF, the subsequent step involves constructing a table that encompasses all available ICF code qualifiers for each individual. To facilitate the comparison of different individuals' time series, the date of a person's initial qualifier is consistently defined as 0. Each successive measurement day for the individual is then assigned a number $d\mathbb{\in N}$, representing the number of full calendar days since the commencement of the treatment. We can then define $\text{TE}$, the time elapsed in days from the most recent valid qualifier, as:

$$\begin{aligned} \text{TE}=d-d_{0},\#\left( e3 \right) \end{aligned}$$

where $d_{0}$ denotes the date of the individual's most recent valid qualifier, excluding *d*. The raw time weighting $\alpha$ is subsequently defined as:

$$\begin{aligned} \alpha=\gamma^{\text{TE}},\#\left( e4 \right) \end{aligned}$$

where $\gamma\in\left( 0,1 \right]$ represents the time decay constant, defined by the user prior to the computation of the index. Consequently, a lower $\gamma$ signifies a stronger time decay. This approach draws inspiration from ^2^.

## Normalization of the time weighting, linkage reliability and $\beta$ term

To ensure comparability between different time weightings and reliability values, we compute the normalized time-weighting values $\tilde{\alpha}_{mi}$ and $\tilde{\alpha}_{\mathrm{sj}}$, normalized reliability values $\tilde{r}_{\mathrm{mi}}$ and $\tilde{r}_{sj}$, as well as normalized $\beta$ term values $\tilde{\beta}_{mi}$ and $\tilde{\beta}_{\mathrm{sj}}$, as follows:

$$\begin{aligned} \tilde{\alpha}_{mi}=\frac{\alpha_{mi}}{\sum_{i\in D} \alpha_{mi}+\sum_{k\in ch_{q}} \sum_{j\in k} \alpha_{sj}+\sum_{k\in ch_{q}} \alpha_{xk}}\#\left( e5 \right) \end{aligned}$$

and

$$\begin{aligned} \tilde{\alpha}_{sj}=\frac{\alpha_{sj}}{\sum_{i\in D} \alpha_{mi}+\sum_{k\in ch_{q}} \sum_{j\in k} \alpha_{sj}+\sum_{k\in ch_{q}} \alpha_{xk}},\#\left( e6 \right) \end{aligned}$$

where $\alpha$ represents the raw, non-normalized time-weighting that we defined in Eq. e4. Furthermore, the normalized reliability values and normalized $\beta$ term values are computed in an identical manner, by substituting the $\alpha$ terms in the equations with *r* or $\beta$, respectively. Because of this normalization, the sum of all normalized terms equals one.

# References

2. Baruah RD, Angelov P, Baruah D. Dynamically evolving clustering for data streams. In: *2014 IEEE Conference on Evolving and Adaptive Intelligent Systems (EAIS)*. ; 2014:1-6. doi:10.1109/EAIS.2014.6867473
